# A Contig-Based Strategy for the Genome-Wide Discovery of MicroRNAs without Complete Genome Resources

**DOI:** 10.1371/journal.pone.0088179

**Published:** 2014-02-07

**Authors:** Jun-Zhi Wen, Jian-You Liao, Ling-Ling Zheng, Hui Xu, Jian-Hua Yang, Dao-Gang Guan, Si-Min Zhang, Hui Zhou, Liang-Hu Qu

**Affiliations:** 1 Key Laboratory of Gene Engineering of the Ministry of Education, State Key Laboratory of Biocontrol, and School of Life Sciences, Sun Yat-sen University, Guangzhou, P. R. China; 2 Guangdong Provincial Key Laboratory of Malignant Tumor Epigenetics and Gene Regulation, Research Center of Medicine, Sun Yat-Sen Memorial Hospital, Sun Yat-sen University, Guangzhou, P. R. China; New England Biolabs, Inc., United States of America

## Abstract

MicroRNAs (miRNAs) are important regulators of many cellular processes and exist in a wide range of eukaryotes. High-throughput sequencing is a mainstream method of miRNA identification through which it is possible to obtain the complete small RNA profile of an organism. Currently, most approaches to miRNA identification rely on a reference genome for the prediction of hairpin structures. However, many species of economic and phylogenetic importance are non-model organisms without complete genome sequences, and this limits miRNA discovery. Here, to overcome this limitation, we have developed a contig-based miRNA identification strategy. We applied this method to a triploid species of edible banana (GCTCV-119, *Musa spp.* AAA group) and identified 180 pre-miRNAs and 314 mature miRNAs, which is three times more than those were predicted by the available dataset-based methods (represented by EST+GSS). Based on the recently published miRNA data set of *Musa acuminate,* the recall rate and precision of our strategy are estimated to be 70.6% and 92.2%, respectively, significantly better than those of EST+GSS-based strategy (10.2% and 50.0%, respectively). Our novel, efficient and cost-effective strategy facilitates the study of the functional and evolutionary role of miRNAs, as well as miRNA-based molecular breeding, in non-model species of economic or evolutionary interest.

## Introduction

MicroRNAs (miRNAs) are single-stranded non-coding RNAs (ncRNAs) that range in size from approximately 20 to 22 nucleotides (nt) and are produced from the cleavage of short RNA hairpins by a conserved RNase III known as Dicer in animals and Dicer-like1 (DCL1) in plants [1]–[3]. miRNAs exist in a wide range of multicellular eukaryotes and in some unicellular eukaryotes, such as *Chlamydomonas reinhardtii* [4], [5]. Studies of miRNAs in well-known model organisms, e.g., mouse, human, rice and *Arabidopsis,* have revealed that these molecules function in almost all important biological processes, including development, metabolism, pathogenic response and apoptosis [3],[6]–[13]. As their functional importance in eukaryotes, miRNAs have become a major research focus in molecular biology.

miRNAs represent a large and diverse family of non-coding genes. Although some miRNAs are highly conserved throughout evolution, a large proportion are newly evolved in each species and species-specific [14]. Thus, organisms can have overlapping but significantly different miRNA profiles. A large number of miRNAs have been identified from a range of organisms: miRBase (v19) [14] contains 21264 miRNAs from 193 species, which represents only a very small proportion of all known species.

Currently, the simplest and most efficient method to identify miRNAs on a genome-wide scale is to perform deep sequencing small RNA (sRNA) transcriptome [15]. Deep sequencing can generate the sequences of almost all types of sRNAs encoded in the genome, including all mature miRNAs. In this type of miRNA identification study, the core challenge is to discriminate mature miRNA sequences from the tens of thousands of small RNA sequences with similar features such as sequence length, nucleotide distribution and genomic localization. However, miRNA genes have a prominent characteristic to distinguish them from other sRNA genes i.e., they have short hairpin structures, and it is therefore easy to resolve this challenge if the species of interest has genomic sequence resources, from which the miRNA precursor sequences may be extracted. Many deep sequencing data- and miRNA precursor sequence-based miRNA identification algorithms have been successfully developed to automatically identify miRNAs from sRNA transcriptomes, for example, miRDeep for animals [16] and mirExplorer for animals and plants [17]. However, the challenge is greater in non-model organisms because of the lack of available genome data.

Several strategies have been proposed to resolve the challenge of miRNA identification in non-model organisms. One such strategy is based on homology searching [18] : this method can identify only conserved miRNAs, which represent just a small portion of the whole miRNA profile, and it cannot identify miRNA precursors which are also important for their functional study. Another strategy is to find a substitute for the genome sequence that can be used to extract miRNA precursor sequences. The genome sequence of a sibling species can be used, but not all non-model species have sequenced siblings, and species-specific miRNAs cannot be identified using this approach. The most frequently used genome sequence substitutes are expressed sequence tag (EST) and genome survey sequence (GSS) libraries, due to their availability in GenBank [19]–[22]. Thus, available dataset-based methods are in most case represented by EST+GSS[20], [23]–[26]. These sequences only represent a very small proportion of genome sequences [27], [28], and they are not specifically designed to include miRNA precursors. Thus, this strategy does not produce optimal results. Moreover, as with the approach of using the genome sequence of a sibling species, not all non-model species have EST or GSS data or other appropriate genome sequence substitutes. This restricts the application of this strategy to the small number of non-model organisms that have genome sequence substitutes available.

In this study, we have developed a novel, systematic and cost-effective strategy based on short DNA contigs with the deep sequencing of small RNA transcriptomes to identify miRNAs in species without completed genome resources. The application of this strategy to the identification of miRNA sequences in the triploid edible banana (GCTCV-119, *Musa spp.* AAA group) demonstrates that our approach can effectively improve miRNA identification in non-model organisms. We suggest that our strategy can promote the study of miRNA in non-model organisms, many of which are species of phylogenetic or economical importance.

## Results

### Strategy for the Genome-wide Identification of miRNAs

**Figure 1:**
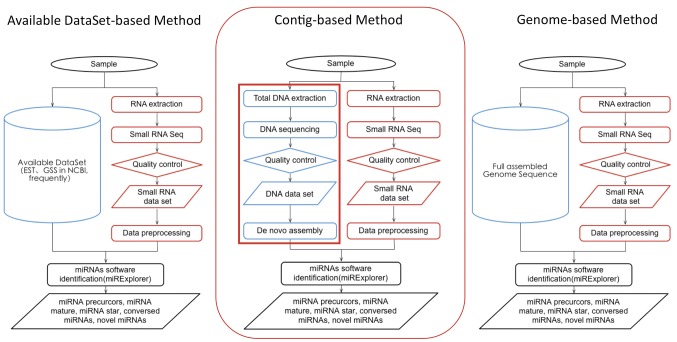
Workflow of the contig-based strategy with current mainstream method for miRNA identification. Acquiring candidate miRNA precursors for hairpin structures is the first step in miRNA identification. This strategy is based on contigs from genomic DNA sequencing, replacing available dataset-based methods (represented by EST+GSS). **Blue-flow**. Pipeline of DNA sequencing **[It is the innovation of this strategy]**; **Red-flow**. Pipeline of small RNA sequencing.

**Figure 2:**
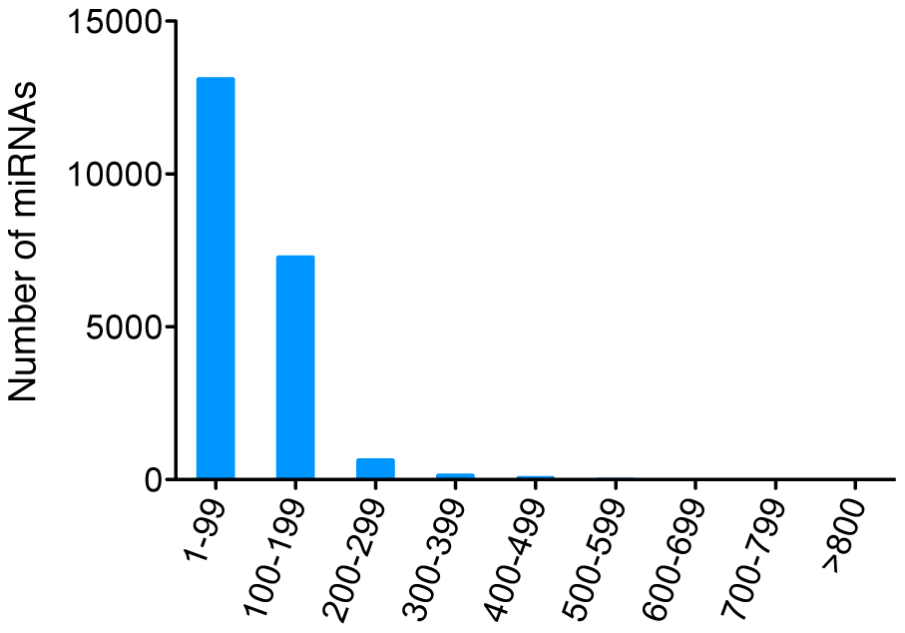
The length distribution of miRNA precursors in the miRBase Release 19. Most miRNA precursors were smaller than 200 nt (95.9%), with only a few over 500 nt (0.1%, most of which are plant miRNAs).

In this study, we have designed a contig-based strategy for the genome-wide identification of miRNAs specifically for use in studies of species lacking genome resources (Figure 1). Our strategy is based on contigs of genomic DNA sequencing with transcriptome sequencing of small RNAs; because of a discovery that length of miRNAs precursors is generally no more than 500-nt (Figure 2) and so the contigs assembled are sufficient. The strategy includes three main steps: 1. Sequencing of genomic DNA and assembly of short contigs; 2. Deep sequencing of the small RNA transcriptome; and 3. mirExplorer identification of miRNA, based on short contig and small RNA transcriptome data. We tested this strategy using a triploid species of edible banana (GCTCV-119, *Musa spp*. AAA group), whose genome has not yet been sequenced.

### Genomic DNA Sequencing and Short Contigs Assembly

We used paired-end sequencing to obtain the genomic DNA sequence of triploid edible banana leaves at a depth of 10-fold, calculated according to the estimated genome size of 600 Mbp, using an Illumina Genomic Analyzer II®. In total, we obtained 94,962,338,100 bp high quality paired-end sequence reads (Figure S1 in File S1).

**Figure 3:**
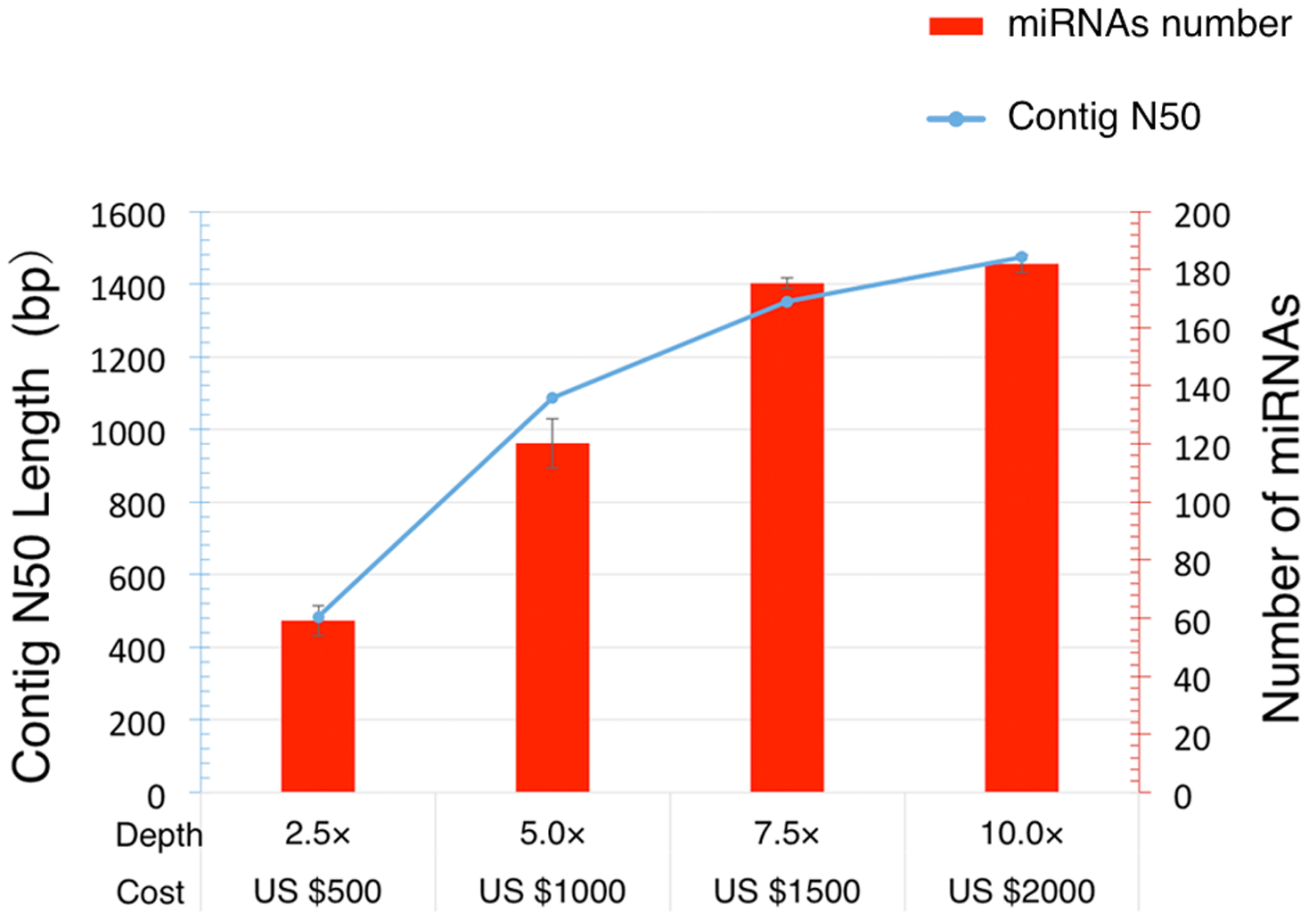
The performance of the contig-based strategy with different depth (cost). Both contig N50 (blue) and the number of identified miRNA (red) increase with depths of DNA sequencing while growth rates decrease. (Cost: the price of DNA sequencing in China, 2011).

Sequence reads were filtered as described in the Methods, and the remaining reads were used for *de novo* assembly. The assembly produced 345,261 contigs, with an N50 size of 1,468 nt and average size of 800 nt (Table 1). We then analyzed the length distribution of all miRNA precursors in miRBase (release 19), including those from animals, plants and viruses. Most miRNA precursors were smaller than 200 nt (95.9%) (Figure 2) with only a few over 500 nt (0.1%), most of which were from plants. This is significantly shorter than the N50 size and the average size of the contigs we obtained, suggesting that our contigs are sufficient for use in the identification of miRNA precursors. Besides, we evaluated the relationship between the contig-based strategy performance (the number of miRNAs identified and contig N50) and cost (depth) of DNA sequencing (Figure 3). The performance gets better as sequencing depth increase, while the growth rates decrease.

**Table 1:** Contigs from the *de novo* Assembly of the Banana (*Musa sp.* AAA) Genome obtained by DNA Genomic Sequencing.

Parameter	Value (nt)
N75	560
N50	1,468
N25	3,245
Maximum	103,591
Average	800

The assembly resulted in a total of 345,261 contigs with an N50 size of 1,468 nt and average size of 800 nt; both values are longer than the sizes of most miRNA precursors in miRBase V19 (500 nt).

### Deep Sequencing of the Small RNA Transcriptome of Triploid Edible Banana Leaves

**Figure 4:**
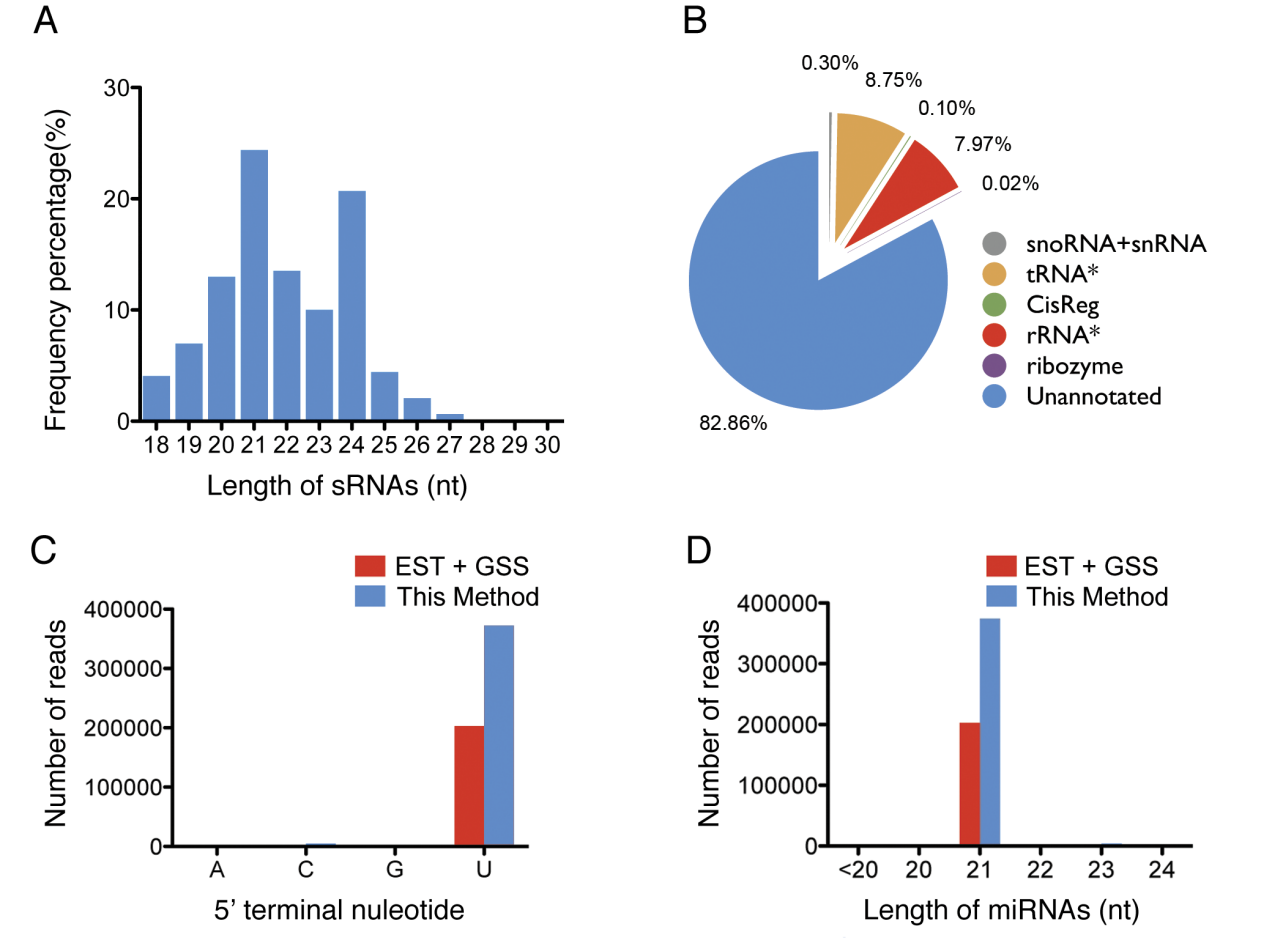
Features of the small RNA sequencing data set. A. Distribution of the length of the small RNA sequencing reads; B. Composition of the small RNA transcriptome of Triploid Edible Banana. (* tRNA &rRNA allow no more than 2 mismatches); C. 5′ terminal nucleotide distribution of mature miRNAs identified by the contig-based and available dataset-based (represented by EST+GSS) strategies; D. Length distribution of mature miRNAs identified by contig-based and available dataset-based (represented by EST+GSS) strategies.

Next, we performed deep sequencing in the small RNA transcriptome of the banana leaves and obtained 10,182,201 reads. After filtering of unusable reads such as low-quality reads, 3′ adaptor-null reads and insert-null reads, 9,703,596 usable reads were obtained (Table 2). The distribution of small RNA lengths was similar to those reported in leaves of other plants (Figure 4A), indicating that these different species may have similar small RNA transcriptome compositions. We annotated the sRNAs by comparing their sequences to the Rfam database (http://rfam.sanger.ac.uk/) (Table S1 in File S1). To reduce the interference of known ncRNA fragments in the miRNA identification process, all sRNAs aligned to known ncRNAs, such as transfer RNA (tRNA, 8.75%), ribosomal RNA (rRNA, 7.97%), small nucleolar RNAs and small nuclear RNA (snoRNA/snRNA, 0.30%), were removed (Table S1 in File S1). The remaining 82.86% of small RNAs from triploid edible banana were unannotated and were retained for miRNA identification (Figure 4B).

**Table 2:** Statistical analysis of small RNA Sequencing in the Triploid Edible Banana (*Musa sp.* AAA).

Type of Reads	Count
total reads	10182201
high quality	10035202
3'adapter null	30059
insert null	2982
5'adapter contaminants	19356
smaller than 18 nt	279095
polyA	114
clean reads	9703596

We obtained 10,182,201 reads from deep sequencing of the small RNA transcriptome of triploid edible banana leaves. After filtering out unusable reads (e.g., low quality reads, 3′ adaptor null reads and insert null reads), 9,703,596 reads were usable.

### miRNA Identification Based on Short Contig and Small RNA Transcriptomic Data

**Figure 5:**
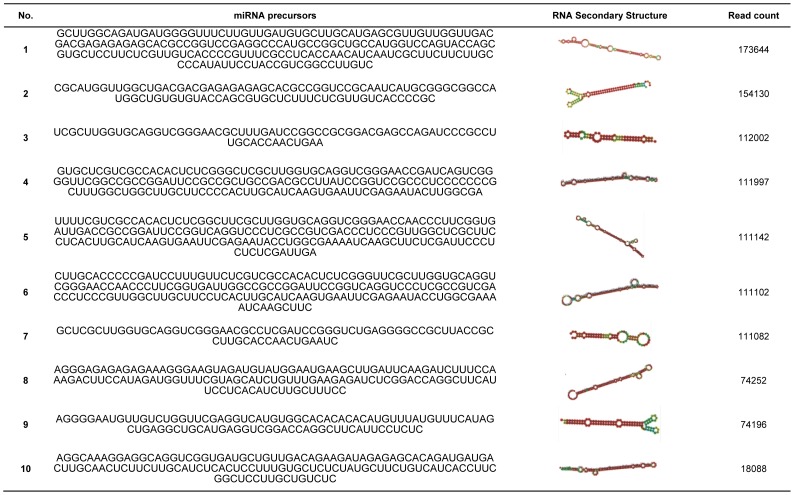
The top 10 most abundant miRNAs identified by contig-based strategy.

We then used our previously developed software mirExplorer to identify triploid edible banana miRNAs. mirExplorer is a machine learning program that can precisely identify plant and animal miRNAs when both the genomic DNA and small RNA transcriptome data are available [17]. In total, we found 314 mature miRNAs corresponding to 180 pre-miRNAs (Table S2 in File S1). The 10 miRNAs with highest read counts are presented in Figure 5. Analysis of the sequence characteristics of the mature miRNAs that we identified show that they are similar to canonical plant miRNAs; i.e., the majority have a uridine at the 5′ end (Figure 4C) and are 21 nt in length (Figure 4D), implying that they are bona fide miRNAs. Considering that miRNAs have tissue-specific expression and we only used leaf sRNA transcriptome data, our triploid edible banana miRNA profile may not be complete. We then analyzed the conservation of pre-miRNAs identified, of which 114 are functional miRNAs annotated in other plants in miRBase and 66 novel miRNAs are identified. The ratio (114/180 = 63.3%) is close to the percentage (60%) of conserved pre-miRNAs of miRNAs in other known species [19], [22], [23], [29]. Therefore, our method has a good performance to identify both conserved and novel miRNAs.

### Comparison of the Contig-based miRNA Identification Strategy to the EST+ GSS-based Strategy

**Figure 6:**
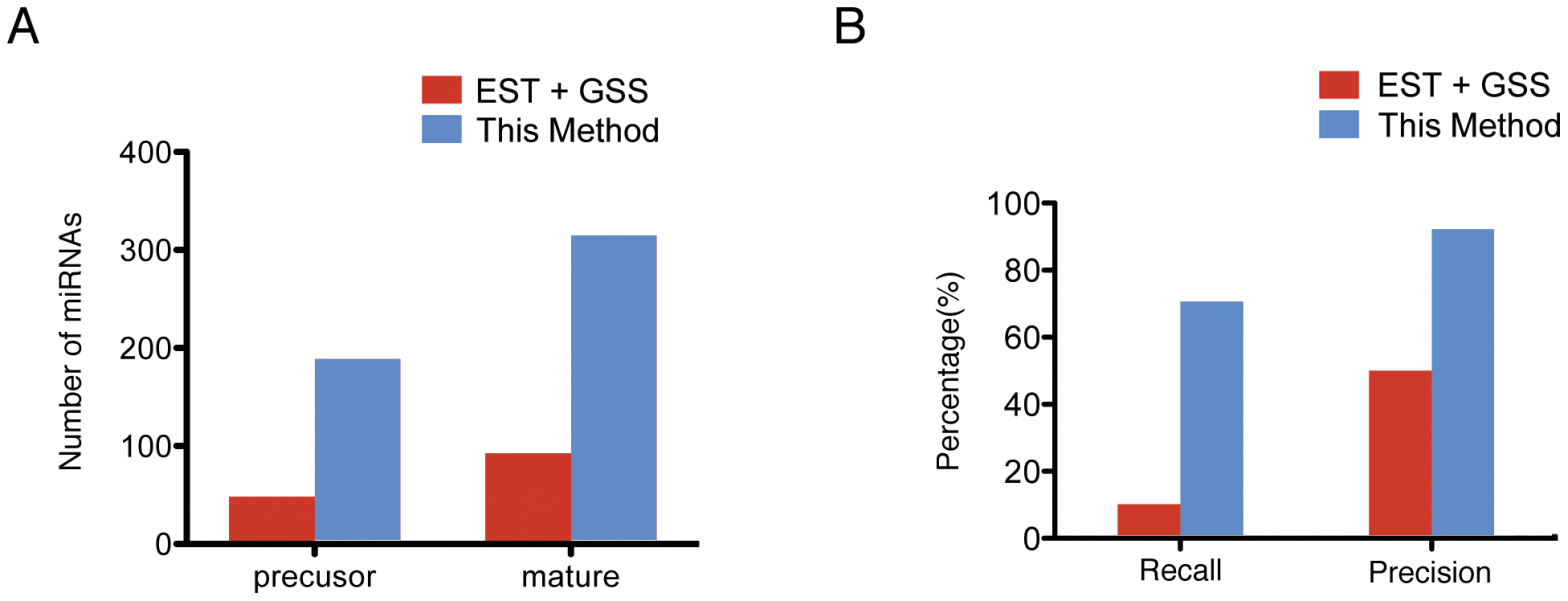
Comparison of this strategy with the available dataset-based methods (represented by EST+GSS). A. The number of mature miRNAs identified by this method and the available dataset-based methods (represented by EST+GSS); B. Evaluation of this method and the available dataset-based methods (represented by EST+GSS), compared with the whole-genome result.

As mentioned above, having available miRNA precursor candidate sequence information is indispensable for the identification of miRNAs. Thus, many previous studies have used EST and/or GSS sequences (depending on whether they were available) as a substitution. As both EST and GSS sequences were available for our test species, we compared the performance of the EST+GSS-based strategy and our contig-based strategy in the identification of miRNAs. We used mirExplorer to identify a total of 97 mature miRNAs corresponding to 52 pre-miRNAs; these are both less than one third of the number of miRNAs identified from short contig sequences (Figure 6A).

While our study was underway, the Global *Musa* Genomics Consortium (GMGC, http://www.musagenomics.org/) published the genome of a sibling banana species, *Musa acuminata* doubled-haploid genotype, including annotation of the predicted miRNA sequences. The species we used is banana GCTCV-119 (AAA, 3n = 33) and the GMGC genome data for comparison of the effectiveness is from banana DH-Pahang (AA, 2n = 22), they are the same species with repeated polyploidization. Because miRNAs are well conserved in eukaryotic organisms, miRNAs in the same species do not change significantly. Thus, we choose GMGC genome (which is the only available genome resource in banana) to benchmark this method. The total number of miRNA genes annotated by GMGC was 235 [30], from which 70.6% were recoverable using our contig-based miRNA identification strategy on only leaves sRNA transcriptome data. Considering the spatio-temporal expression pattern of miRNAs, this result supports the high identification power of the contig-based miRNA identification approach. In contrast, the available dataset-based (represented by EST+GSS) strategy recovered only 10.2% of the GMGC miRNAs. We then assessed the prediction precision of these two miRNA identification strategies. The contig-based strategy achieved a high precision rate of 92.2%, while the available dataset-based (represented by EST+GSS) strategy had a precision rate of only 50.0% (Figure 6B). These results indicate that performance of the contig-based miRNA identification strategy is much better than that of the available dataset-based (represented by EST+GSS) strategy.

## Discussion

The identification of miRNA is the first step in understanding its function, and acquiring candidate miRNA precursors for hairpin structure prediction is the first step of this process [31]. Generally, the identification of these candidates relies on extracting their sequence from the entire genome. However, many species of economic or phylogenetic interest are non-model organisms and do not have complete genome resources. Thus, the identification of miRNAs in non-model organisms can be a great challenge.

Previously, the identification of miRNA in non-model organisms made frequent use of EST and/or GSS sequences, if accessible, as a popular substitution for an available genomic sequence [20],[23], [32]–[34]. An EST library, reflecting the cDNA in technical principle, only represents a very small proportion of genome sequences [27], [28] and is not specifically designed to capture miRNA precursor sequences. GSSs are nucleotide sequences similar to ESTs, with the exception that most are of origin from genome rather than miRNA. There are several drawbacks to the use of ESTs for the identification of miRNAs. First, not all organisms have EST and/or GSS resources. Second, the quality and quantity of EST and GSS data of different organisms can vary, and so the miRNA identification will be limited by data of the species being studied, which is beyond the control of investigators. Third, most miRNA precursors have low cellular abundance and have, therefore, only a low probability of being sequenced in EST experiments. Furthermore, it has been discovered that approximately 40% of all known miRNAs are encoded within intronic sequences [35], [36], and this subset of miRNAs might not be sequenced. If the intact pre-miRNA sequences were not present in the EST data set, the corresponding miRNAs would not be identified if miRNA identification were solely based on EST sequences. Therefore, it should be expected that EST and/or GSS sequence-based miRNA identification will not be comprehensive. Consistent with this hypothesis, the total number of miRNAs identified by the available dataset-based (represented by EST+GSS) strategy was 70% less than were identified by the contig-based strategy.

Although whole-genome sequencing technology has made great progress, and its cost has decreased dramatically, the *de novo* sequencing of a genome is still not an easy task and requires many complex steps. For example, scaffolding is an important step in acquiring the whole genome; scaffolds are composed of contigs and gaps, and used to reconstruct entire chromosomes. Scaffolding requires different strategies for building of several DNA libraries and is expensive and time consuming [37]. However, gaps in the scaffolds are filtered during this process and so cannot be used in miRNA identification. Distinguishing each genomic repeat region requires the combination of multiple sequencing techniques, even when Sanger sequencing is used [38]. These steps are also expensive and time-consuming. Most miRNA precursors are very short in length (<200-nt); it is therefore possible to use DNA contigs as pre-miRNA candidates for the identification of miRNAs. Deep sequencing technology makes it easy to obtain short contigs covering most genomic regions of the species of interest. Moreover, using short contigs is also cost and time efficient because their sequencing can be finished in one experiment. In this study, we have sequenced the triploid edible banana genome at a coverage of only 10-fold and have constructed contigs with an N50 size of 1,468 bp and average size of 800 bp (Table 1); these lengths are significantly longer than known miRNA precursors. Finally, we successfully identified a large number of triploid edible banana miRNAs from the assembled short contigs, supporting the feasibility of our contig-based miRNA identification strategy.

For finding a minimal requirement to achieve similar results that another user could use as a guide if they wanted to take this approach. We have made the analysis on coverage and contig size requirement. The result showed that both contig N50 and the number of identified miRNA grown with depth of DNA sequencing with a decreased growth rates. And 7.5×depth (cost about US $1500 in Chinese price of 2011) strikes a good balance between performance and cost. While small RNA sequencing is not expensive, so we use default small RNA sequencing (1 GB).

In this study, we have designed an efficient and cost-effective strategy for the *de novo* identification of miRNAs in non-model organisms that do not have reference genomes. miRNAs represent a large and important family of non-coding genes that exists widely in plants, animals and viruses, and they have species-specific and spatio-temporally specific expression patterns. miRNAs are involved in almost all important biological processes, such as development, metabolism, pathogen response and apoptosis [39]. For many non-model species of economic importance, such as coffee, tea, ginkgo and *Taxus*, miRNAs can be key to the improvement of a trait [40] in a particular breed. Regardless of economic importance, the question of how miRNAs originated, and have evolved, in eukaryotes is of interest. However, unlike higher organisms, most primitive organisms do not have completed genome resources, meaning that the identification of miRNA in these species is difficult. Furthermore, it remains unclear whether miRNAs existed universally in early protists and what roles they played in these ancestral organisms [41]–[43]. To answer these questions, it is necessary to identify miRNAs in a wide range of phylogenetic species. Thus, our method provides an easy way to obtain the data necessary for these studies relating to miRNA in many different non-model organisms.

In conclusion, we have developed an efficient and cost-effective strategy for the identification of miRNAs in non-model organisms. We applied this strategy to triploid edible banana miRNA and identified 314 mature miRNAs and 180 pre-miRNAs. Although we mainly focus on miRNA identification in this study, our strategy may also be applied to the identification dependent on genome resources of other small ncRNAs, such as siRNAs. This strategy is efficient for the genetic study and molecular breeding of economically important species and for phylogenetic research on the origin and evolution of miRNAs, which often involves species that lack well developed genome resources.

## Materials and Methods

### Plant Material

The leaves of edible banana (GCTCV-119, *Musa spp*. AAA) were provided by the Guangdong Academy of Agricultural Sciences.

### Genomic DNA Isolation, Sequencing and Assembly

Total DNA was extracted from banana leaves using the E.Z.N.A. ® HP Plant DNA Kit (Omega®, D2485–00), according to the manufacturer’s protocol. The concentration and purity of total DNA were assessed using a NanoDrop® spectrophotometer. For the sequencing of the triploid edible banana genome, a 500 bp DNA insert library was constructed from the samples and was deep sequenced using Illumina® Genome Analyzer II. To control the quality of the raw data, the genomic DNA sequence reads were processed using the bioinformatics pipeline FastQC (http://www.bioinformatics.babraham.ac.uk/projects/fastqc/), as follows: 1) removed low quality reads; 2) removed adaptor contaminants formed by adaptor and adaptor ligation; and 3) trimmed 3′ prime adaptor sequences.

To obtain contigs for miRNA precursors using *de novo* assembly, short read assemblers such as SOAPdenovo (http://soap.genomics.org.cn/soapdenovo.html) were used. These assemblers are based on a *De bruijn* graph approach and widely used for the *de novo* assembly of short paired-end reads generated by the Illumina Genome Analyzer. All assemblers were run using default parameters.

### RNA Processing for miRNA Prediction

Total RNA was isolated with the Plant/Fungi Total RNA Purification Kit (Norgen Cat# 25800, 31300, 31900), as described in the manufacturer’s instructions. RNA concentrations and purity were determined spectrophotometrically using a NanoDrop® spectrophotometer. A small RNA library was built with small RNAs isolated from total RNA samples (mostly 18∼30-nt) through adaptor ligation, purification and reverse transcription. The small-RNA library was sequenced with an Illumina® Genome Analyzer II. All reads from the RNA library were processed with the FastQC pipeline, as previously described.

To find known ncRNAs such as tRNAs, rRNAs, and snoRNAs, we searched the Rfam database [44] (http://www.sanger.ac.uk/software/Rfam) and the GenBank noncoding RNA database (http://www.ncbi.nlm.nih.gov/).

### miRNA Identification

In this study, we used the software mirExplorer for identification of miRNA [17]. It is able to identify miRNAs with NGS data from a wide range of species, including plants. Using mirExplorer, miRNAs were identified from miRNA precursors obtained by deep sequencing, and from mature miRNAs obtained by small RNA sequencing. An optimized mirExplorer is for our novel method. We modified two parameters of the program, extending 55 bases upstream and 165 bases downstream, while the other parameters were set to the defaults.

### Evaluation of our Strategy and the Available Dataset-based Method (Represented by EST+GSS)

To investigate the efficiency of our method compared with the available dataset-based methods, which is in most case represented by EST+GSS, we collected datasets as follows:

EST+GSS data from banana were obtained from the National Center for Biotechnology Information (NCBI, http://www.ncbi.nlm.nih.gov/); to date, 32419 ESTs and 31465 GSSs from banana can be obtained from this source.

During our research, the sequence of one banana genome was published in *Nature*, offering us a baseline for comparison of the effectiveness of these strategies (Accession number: HE813975–HE813985) [30]. In the Global *Musa* Genomics Consortium study, 235 miRNAs were identified from the miRNA precursors predicted in the genome. Total miRNAs predicted by whole-genome sequencing were downloaded from the article.

To evaluate the performance levels of the two methods, the values for recall rate (R) and precision (P) were calculated using the following equations [45] :

Recall = true positive/(true positive+false negative).

Precision = true positive/(true positive+false positive).

## Supporting Information

File S1 **Figure S1, FastQC evaluation of the quality of genomic DNA and small RNA sequencing results. Table S1, Distribution of the various types of small RNAs in Banana (AAA). Table S2, List of all 180 miRNAs identified by the contig-based method.**
(DOC)
