# Supplementary material for: A Contig-Based Strategy for the Genome-Wide Discovery of MicroRNAs without Complete Genome Resources
Source: PLoS One. 2014 Feb 7;9(2):e88179. doi: 10.1371/journal.pone.0088179 (PMC3917882; doi:10.1371/journal.pone.0088179)
Supplement: File S1 — Figure S1, FastQC evaluation of the quality of genomic DNA and small RNA sequencing results. Table S1, Distribution of the various types of small RNAs in Banana (AAA). Table S2, List of all 180 miRNAs identified by the contig-based method. (DOC) [file pone.0088179.s001.doc]

**Figure S1. FastQC evaluation of the quality of genomic DNA and small RNA sequencing results.**


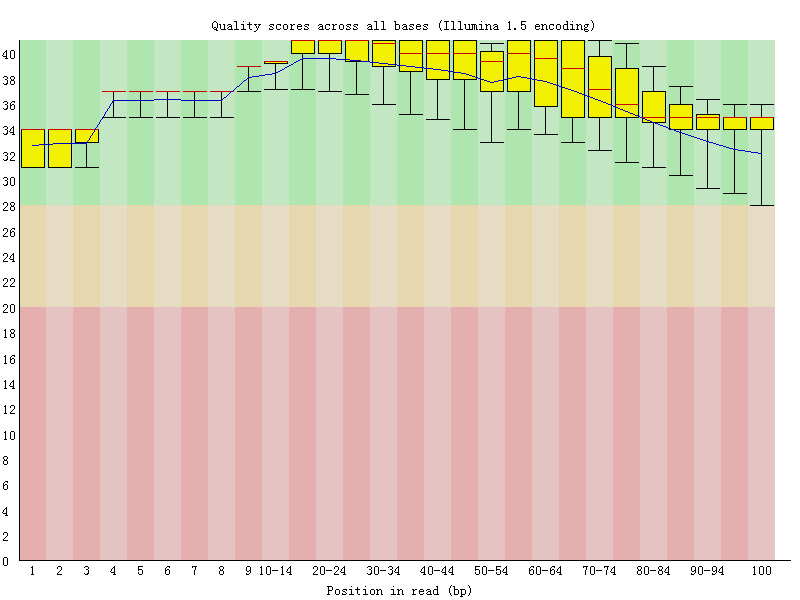

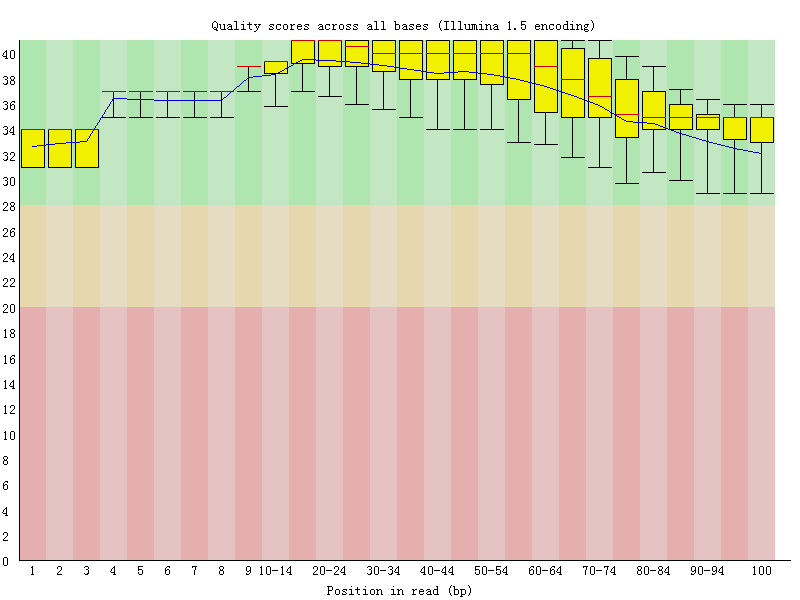


Genomic DNA sequencing: Paired-end 1

Genomic DNA sequencing: Paired-end 2


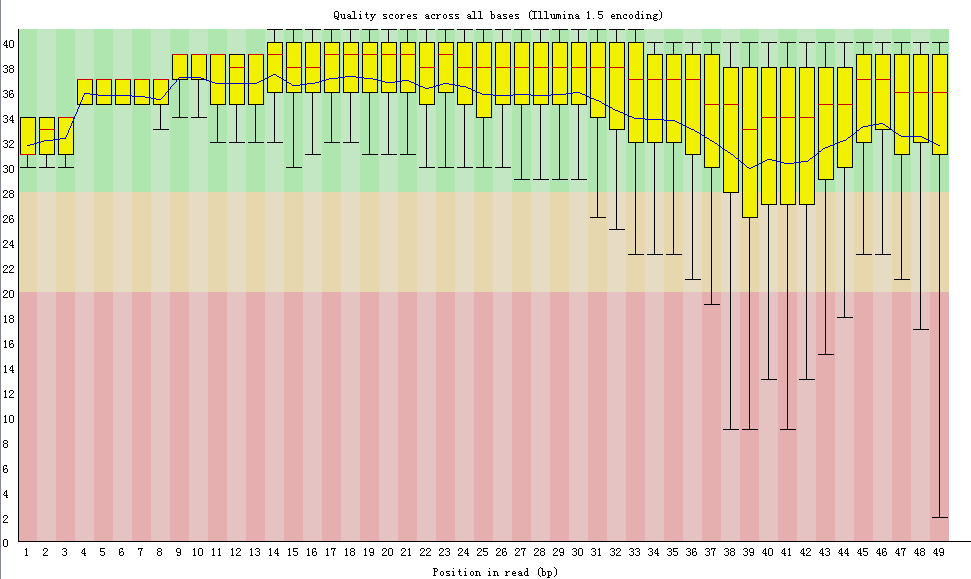


Small RNA sequencing

**Table S1. Distribution of the various types of small RNAs in Banana (AAA).**

| Category | Unique RNAs | Percent | Total RNAs | Percent |
| --- | --- | --- | --- | --- |
| Total small RNAs | 1627622 | 100.00% | 9982805 | 100.00% |
| snoRNA+snRNA | 4888 | 0.30% | 37567 | 0.38% |
| tRNA* | 142419 | 8.75% | 1541412 | 15.44% |
| rRNA* | 129793 | 7.97% | 1002693 | 10.04% |
| CisReg | 1632 | 0.10% | 4329 | 0.04% |
| ribozyme | 292 | 0.02% | 707 | 0.01% |
| Unannotated | 1348598 | 82.86% | 7396097 | 74.09% |

Notes:* tRNA &rRNA are allowed no more than 2 mismatches.

**Table S2. List of all 180 miRNAs identified by the contig-based method.**

| No. | miRNA precursors | miRNA mature | miRNA star |
| --- | --- | --- | --- |
| 1 | GCUUGGCAGAUGAUGGGGUUUCUUGUUGAUGUGCUUGCAUGAGCGUUGUUGGUUGACGACGAGAGAGAGCACGCCGGUCCGAGGCCCAUGCCGGCUGCCAUGGUCCAGUACCAGCGUGCUCCUUCUCGUUGUCACCCCGUUUCGCCUCACCAACAUCAAUCGCUUCUUCUUGCCCCAUAUUCCUACCGUCGGCCUUGUC | UGACGACGAGAGAGAGCACGC | GUGCUCCUUCUCGUUGUCACC |
| 2 | CGCAUGGUUGGCUGACGACGAGAGAGAGCACGCCGGUCCGCAAUCAUGCGGGCGGCCAUGGCUGUGUGUACCAGCGUGCUCUUUCUCGUUGUCACCCCGC | UGACGACGAGAGAGAGCACGC | GUGCUCUUUCUCGUUGUCACCC |
| 3 | UCGCUUGGUGCAGGUCGGGAACGCUUUGAUCCGGCCGCGGACGAGCCAGAUCCCGCCUUGCACCAACUGAA | UCGCUUGGUGCAGGUCGGGAA | CCCGCCUUGCACCAACUGAA |
| 4 | GUGCUCGUCGCCACACUCUCGGGCUCGCUUGGUGCAGGUCGGGAACCGAUCAGUCGGGGUUCGGCCGCCGGAUUCCGCCGCUGCCGACGCCUUAUCCGGUCCGCCCUCCCCCCCGCUUUGGCUGGCUUGCUUCCCCACUUGCAUCAAGUGAAUUCGAGAAUACUUGGCGA | UCGCUUGGUGCAGGUCGGGAA | CCCCACUUGCAUCAAGUGAAU |
| 5 | UUUUCGUCGCCACACUCUCGGCUUCGCUUGGUGCAGGUCGGGAACCAACCCUUCGGUGAUUGACCGCCGGAUUCCGGUCAGGUCCCUCGCCGUCGACCCUCCCGUUGGCUCGCUUCCUCACUUGCAUCAAGUGAAUUCGAGAAUACCUGGCGAAAAUCAAGCUUCUCGAUUCCCUCUCUCGAUUGA | UCGCUUGGUGCAGGUCGGGAA | ACUUGCAUCAAGUGAAUUCGAGAA |
| 6 | CUUGCACCCCCGAUCCUUUGUUCUCGUCGCCACACUCUCGGGUUCGCUUGGUGCAGGUCGGGAACCAACCCUUCGGUGAUUGGCCGCCGGAUUCCGGUCAGGUCCCUCGCCGUCGACCCUCCCGUUGGCUUGCUUCCUCACUUGCAUCAAGUGAAUUCGAGAAUACCUGGCGAAAAUCAAGCUUC | UCGCUUGGUGCAGGUCGGGAA | ACUUGCAUCAAGUGAAUUCGAGAA |
| 7 | GCUCGCUUGGUGCAGGUCGGGAACGCCUCGAUCCGGGUCUGAGGGGCCGCUUACCGCCUUGCACCAACUGAAUC | UCGCUUGGUGCAGGUCGGGAA | CCGCCUUGCACCAACUGAAUC |
| 8 | AGGGAGAGAGAGAAAGGGAAGUAGAUGUAUGGAAUGAAGCUUGAUUCAAGAUCUUUCCAAAGACUUCCAUAGAUGGUUUCGUAGCAUCUGUUUGAAGAGAUCUCGGACCAGGCUUCAUUCCUCACAUCUUGCUUUCC | UCGGACCAGGCUUCAUUCCUC | GAAUGAAGCUUGAUUCAAG |
| 9 | AGGGGAAUGUUGUCUGGUUCGAGGUCAUGUGGCACACACACAUGUUUAUGUUUCAUAGCUGAGGCUGCAUGAGGUCGGACCAGGCUUCAUUCCUCUC | UCGGACCAGGCUUCAUUCCUC | AAUGUUGUCUGGUUCGAGGUC |
| 10 | AGGCAAAGGAGGCAGGUCGGUGAUGCUGUUGACAGAAGAUAGAGAGCACAGAUGAUGACUUGCAACUCUUCUUGCAUCUCACUCCUUUGUGCUCUCUAUGCUUCUGUCAUCACCUUCGGCUCCUUGCUGUCUC | UUGACAGAAGAUAGAGAGCAC | GCUCUCUAUGCUUCUGUCAUC |
| 11 | UUUGAAUCUGUUUUGUUGAUGGUCAUCUAAUCAUUUCCAAAUUCCUCCCUGUCUUGUUGAGUGAGGCCAUGAUUAGAUGACCAUCAGCAAACAAGUUCACAAG | UUAGAUGACCAUCAGCAAACA | UUUUGUUGAUGGUCAUCUAAUC |
| 12 | CGGAGGAGAAAGAUGUGAUUUUAUGUGAGGAGGAGGCGGAGGAGAAUGAGUGGUUGACAGAAGAGAGCGAGCACACGGCGGGGGGAAAUGGUAUGGUAUGAUGCUGUUUCCGUUGCGUGCUCACUUCUCUUCUUGUCAACC | UUGACAGAAGAGAGCGAGCAC | GCUCACUUCUCUUCUUGUCAACC |
| 13 | UCGUCCAUAAGUUCCCGGUUCUUCAGCGCGGAGUAGAGCAGUUUGGUAGCUCGCAAGGCUCAUAACCUUGAGGUCACGGGUUCAAAUCCUGUCUCCGC | CGCGGAGUAGAGCAGUUUGGUAG | AACCUUGAGGUCACGGGUUCA |
| 14 | CUUGAAAGAUGGGUUGUUCAGGGUGGAGAAGCAGGGUACGUGCAUUCCUACCACAUUUCGUCCUCUCAGCGAAGAGUGUGCUGGUUCUUCAUGUGCCCAUCUUCUCCAUCAUGAACAACCGAUCUU | UGGAGAAGCAGGGUACGUGCA | CAUGUGCCCAUCUUCUCCAUC |
| 15 | GAUGGGUUGUUCAGGGUGGAGAAGCAGGGUACGUGCAUUCCAUCCACAUUUCCUCCUCUCAGGGUAGAGUGUGCUGGUUCUUCAUGUGCCCAUCUUCUCCAUCAUGAACGACCGAUCUCGUAUACUGCCAAUCGCAAAGUAUGAUGAGUCGG | UGGAGAAGCAGGGUACGUGCA | CAUGUGCCCAUCUUCUCCAUC |
| 16 | AUGGAGAAGCAGGGUACGUGCAUUACUCGCUAACAUCUAGUCUUCUUCACCUAGUAAACUGAUGGCCAGUUCUUCAUGUGCCCAUCUUCUCCAUC | UGGAGAAGCAGGGUACGUGCA | CAUGUGCCCAUCUUCUCCAUC |
| 17 | AUCUCUGGACGCAGCGGUUUAUCGAUCUCUUCCUGGCCUUGUCGGUCUCUUUUCAUGGUGGAUUAGAUCGGUUCUUUUCUUUUUGAGGUUACAAAGCGGGAAUCGGUCGAUAAACCUCUGCAUCCGGACUUCCGACGA | UCGAUAAACCUCUGCAUCCGG | UGGACGCAGCGGUUUAUCGAU |
| 18 | AGAGGUAUUGGCGUGUCUCAAUCUGAAGACAUGGUUGGAUGUUGAAACAACCAUGUAAUUGGAUUGAGCCGCGUCAAUAUCUCUC | UUGAGCCGCGUCAAUAUCUCU | UAUUGGCGUGUCUCAAUCUGAA |
| 19 | GUUGACAACGAGAGAGAGCACGCCGGUCCGUAAUCAUGCGAGCGGCCAUGGUUGUGUGUACCAGCGUGCUCUUUCUCGUCGUCACC | GUGCUCUUUCUCGUCGUCACC | UGACAACGAGAGAGAGCACGCC |
| 20 | UCCGGAUUUUGAUGACGCCCUCUGAAGUCGCAUGCGUGGUUGACAACGAGAGAGAGCACGCCGGUCCGCAAUCAUGCGGGCGGCCAUGGUUGUGUGUACCAGCGUGCUCUUUCUCGUCGUCACC | GUGCUCUUUCUCGUCGUCACC | GACAACGAGAGAGAGCACG |
| 21 | UCGGGUUGACAACGAGAGAGAGCACGCCGGUCCGCAAUCAUGGGAGCGGCCAUGGCUGUGUGUACCAGCGUGCUCUUUCUCGUCGUCACCUGGCUCGCUCGCCCU | GUGCUCUUUCUCGUCGUCACC | UGACAACGAGAGAGAGCACGCC |
| 22 | CGUUUGAAUGGUCCGGUGAAGUGUUCGAAUCGAGGCGACGGGGGUGGUUCGCCACCUAUGACGUCGCGAGAAGUCUAUUGAACCUUAUCAUUUAGAGG | UGAAUGGUCCGGUGAAGUGUU | AACCUUAUCAUUUAGAGG |
| 23 | AGUAGUGCGAGGGCAGAUGAUAAUCAAAGAUCGCCGGCAUGUUCUCCACAGGCUUUCUUGAACUGUGAACUAUCUGUAAUCCUGUGUGUCUGAGGAGGAAGGUGGACUCUGUUGACAGUUCAAGAAAGUCCUUGGAAAACAUGACACAUCUUU | UCCACAGGCUUUCUUGAACUG | GUUCAAGAAAGUCCUUGGAAA |
| 24 | UCCUCCACAGGCUUUCUUGAACUGUGAAUGCGUGAAGCUGUGUCCUCGGUCGAGAAACCCAGUUCGUCUGUUGAUAGUUCAAGAAAGUCCCUGGAGAACAUGACUGGUCUUUCAGAAU | UCCACAGGCUUUCUUGAACUG | UAGUUCAAGAAAGUCCCUGG |
| 25 | CAAGAUGGUUUCAUAUGGCUGCAUUUGCACCUGCACCUAAAGGGGAGCGGGGUGGCCGUGAAGAAAACGAGUUUACAUCCACCAUGUAGGUGCAGCGGCAAAUGCAACCAUAUGCCAUUUUGG | UGCAUUUGCACCUGCACCUAA | AGGUGCAGCGGCAAAUGCAAC |
| 26 | AGGAUGUUGCUCCGAUCCUGCAGCUCGUUGGUCCAAACAGGGGCCCCACAAUCACACUGAAAAAAGCAGAUAGCCGGAGCGGAUUGUGGGUCCCUCUUGUUUGAACCAAUGGCUGCACCAUCCAUGUAGCUCGCUG | UUGUUUGAACCAAUGGCUGCA | CCUGCAGCUCGUUGGUCCAAAC |
| 27 | AAGAAGGAAAGAAAGAAAGGGCGCCGAGGAGGUAGCAGUAGCAGUGGAAGGGGCAUGCAGAGGAGAGGAGGGAAGGCGAAAGAAAUCCUGUCUCCUGUGCCUGCCCCUCCCACCCCUGCUGUUACGACUCGC | UGGAAGGGGCAUGCAGAGGAG | CCUGUGCCUGCCCCUCCCACC |
| 28 | GGGGCAAGCACGACAAGGAGUUGGGUUUCCAUGUCUAGGAGAGAUGACACCGGCUUCAUGAGAUGGGUCCAGGUUGGUGUGGAUGAAGCUGGUGCCAUCUCUUCUAGACAGGGGAUCU | UAGGAGAGAUGACACCGGCUU | GCUGGUGCCAUCUCUUCUAGA |
| 29 | UUUCCUCCGCAGGAGAGAUGAUGCCGCUACCGUCGGAAAGCUUCUUGGCUCAGUUACUGGGAAGUUUGGGCAGGCAGCGGUAUCAUCUACUGCGCAGGAAACAUGGC | CCGCAGGAGAGAUGAUGCCGCUA | UUGGGCAGGCAGCGGUAUCAUC |
| 30 | UUUCCUUCUGGGGCAGAUGAUUGUGAGAUGGUGCUUUGAGUUGGUUUUGGUUGACAACGAGAGAGAGCACGCUGGUCUGCACUUAUGCAGGAGGACAUGAAUGUGUACUUGCGUGCUUUCUAUCGUUGUCAAUCAUUGACAUGGCGCCAAGCAGCUUGCUUUUGUUCUUAUGGCUCGCAUGCAGGAAAC | GUGCUUUCUAUCGUUGUCAAU | UGACAACGAGAGAGAGCACGCU |
| 31 | CUGGGUGAGAGUGUGAUGUUGGAAUGGCUCAAUCAGAUUUGAUCAUAUUUUAGUUUGAGAUGUUUGAUCUGAUUGAGCCGUGCCAAUAUCACUCUUUGCUUGGU | UGAUUGAGCCGUGCCAAUAUC | UGUUGGAAUGGCUCAAUCAGA |
| 32 | GCUUGUGCAGUAACAAUGGAUGAGGAUAACAUCACCUUGAGAUAGUUACUAUGAUGUCGGCCCGGCUCACUCAGAGUGCAUGCACUACUCAGGCAAUGCAAAGCCCUCAUGCUUUGUUAUCUGAUUGAGCCGUGCCAAUAUCUUAGUACUCUUUCAUCCCUCCAGCUGUCUUC | UGAUUGAGCCGUGCCAAUAUC | UGUCGGCCCGGCUCACUCAGA |
| 33 | GUCGAUGUUUGCGAGUGCAGCAUCAUCAAGAUUCACAUAUUUUCAGCAUCUUUGGCCUCUGAUAUCAUAUGAGAAUCUUGAUGAUGCUGCACCGGCAAUUGGCGACUACGCUCGUCGAGUCUUUU | AGAAUCUUGAUGAUGCUGCAC | CAGCAUCAUCAAGAUUCAC |
| 34 | GAACGAGAUGUUGGCCCGGUUCAAUUAGAAAGUAGUGCUUCUAUCUUACUAGAAGUUCUAUUGUUUUAAUUGAGCCGUGCCAAUAUC | AGAUGUUGGCCCGGUUCAAUU | UUGAGCCGUGCCAAUAUC |
| 35 | AACGAGAUGUUGGCCCGGUUCAAUUAGAAAGUAGUGCUUCUGUCUUACUAGAAGUUCUAUUGUUUUAAUUGAGCCGUGCCAAUAUCACGUUCGUUGCUCGGAGUGG | AGAUGUUGGCCCGGUUCAAUU | UUGAGCCGUGCCAAUAUCACG |
| 36 | CGAGUUAAGCGAGCCAGUUGUGGGGCUUCUCUCCCUUGGCAGGUGACUCAGAGAGAAAGGAGGUAAGGUUGAAUGCAUCGGUGAUGCGUUUCCUCCGAUGAGUCAGUGACCCGCCAAAGGAGACUUGCCCUACGACUGGC | CGCCAAAGGAGACUUGCCCUA | GGCUUCUCUCCCUUGGCAG |
| 37 | CUGGCGAUGGUUUCAUGGUUAAGCGAGCCAGUUCUGGGGUUUCUCUCCUUCGGCAGGCGAUAGAGGAAAGGUCCCAGAUAAGAGAGAGUUGCAUAUAUAUCAAUGGCCUGCCAAAGGAGAGUUGCCCUACGACUGGCUUCAGCUUAGAGAGAGAGAGAGAGAGCGCUAAGCAUCCA | UGCCAAAGGAGAGUUGCCCUA | GGUUUCUCUCCUUCGGCAG |
| 38 | AGGUGGGUUCUGCAGGGUGGAGAAGCAGGGCACGUGCACUGCUUGCACUCCGCUUCCACCAGAGAAGACCGUGCCAGUUCUUCAUGUGCUCGUCUUCUCCACCGUGCACAACCACAUCUU | UCAUGUGCUCGUCUUCUCCAC | UGGAGAAGCAGGGCACGUGCAC |
| 39 | UUUUUCUCCUCCUCUCAUGACAGUUUAGGGGAAUGUUGUCUGGCUCGAAGCCAUUGGAAUGAGAACAGUGUCUUGAUCUAUCCUUAUCGAGAUGAGUGGAUUCGGACCAGGCUUCAUUCCCCUCAACUCAUGGAAUUGUUUGGAGUCAACUGGAAGAG | UUCGGACCAGGCUUCAUUCCC | GGAAUGUUGUCUGGCUCGAAGC |
| 40 | AAAAGUAUUGCAUGUUCUAGUGAUGGCUGGAUGGCUCUUCUUUGUUUCUGAUAGUUGAGGGGAGUGUCGUCUGGUACGAAGCCAUUGGAACAAACAAAGAGCAACUUCUCACUCCAUUUCUUGUGCUGGCAAAUGGUUUCGGACCAGGCUUCAUUCCCCCUAACUAGUGGCAGUGGUUGUUUCUCCGUUAGUUUCUCCACAAGUAAGUUCGUUUC | UUCGGACCAGGCUUCAUUCCC | GAGUGUCGUCUGGUACGAAG |
| 41 | GGGUUGUGCCUGGCUCCCUGUACGCCACACAUGUAGAUCGAUAUCUAUGGGGAGAUUGGCUACUGUGGGUGGCGUGCAAGGAGCCAAGCAUGCCUU | GCGUGCAAGGAGCCAAGCAUG | UGCCUGGCUCCCUGUACGCCA |
| 42 | CCAAGCGAGACUAUAGGCGACAUGUACACCCGUUUCACGGAUGUCGUCAAUAGUCUAAGAGUUCUUGGU | AAGCGAGACUAUAGGCGACAUGUA | UCACGGAUGUCGUCAAUAGUC |
| 43 | UUCUGGAGAAGUUGGGGAUGACGGCCGUGGGGAAGAGGUAACAUGGACUGUGCCUGGCUCCCUGUAUGCCACUCGUGUGGGCGCCAAUCUAUCGAGACUGGCGACUGCGGCUGGCGUGCGAGGAGCCAAGCAUAUCUCUCUCUCUCUCUCUCUCUCUCUCUCUCUCUCAAAC | UGCCUGGCUCCCUGUAUGCCA | GCGUGCGAGGAGCCAAGCAUA |
| 44 | GUAUGGAAGAAUCCGUAAAGCUCAGGAGGGAUAGCGCCAUGGGUAAGUAGGAACUUCUGUUCUGUUUGUCUAAAGGCGCUAUCUAUCCUGAGUUUCAUGGGUUCUUCCUACUCCAGGAGGAA | CGCUAUCUAUCCUGAGUUUCA | UCAGGAGGGAUAGCGCCAUG |
| 45 | GGUAAGAUGGUUUUGCUCAAGACCGCGCAACACUUCCAUAUGACACCGCAUCGCUGUGCUCAUCUAUCCAUCAGCAGAUACCGAUGGAGAAGGCAUGGAAGGAGCGGGCGAUAGGUUUGGACAGCGAUGCGUUGGUUUAUGGCCAGGCUGCGCGGUCCUGCGCAAGAUCCGCCC | UUUUGCUCAAGACCGCGCAAC | UGCGCGGUCCUGCGCAAGAUC |
| 46 | GGGAAUGUCGUCUGGCUCGAGGUUGCUGUGACUGCUUCAUUUUGUGGAAGAGAUCUCGGACCAGGCUUCAUUCCCC | GGAAUGUCGUCUGGCUCGAGG | UCGGACCAGGCUUCAUUCCCC |
| 47 | AAGAGGCUGCAAGGCGAAAAGGAAAGGGAGAUGAUCGGGUCUGUGGAAGCUCUUGGUAUGGGGCUGGGUUGGAAAACCCUACCGCCCCGCCAUGUUUGUCUUAUUACAUGGAGUGAGGUCUUUUCCAAUGCCUCCCAUGCCAAUGGUUUCCAGGCACCCCUCCCUUUCCCUCUCUGAGUCUUUUG | UUUCCAAUGCCUCCCAUGCCAA | GGUAUGGGGCUGGGUUGGAAAAC |
| 48 | GGGAAUGUUGUCUGGCUCGAGGCCUUCGUUUCUCUUACAGAUCUGUUUGAAGAGAUCUCGGACCAGGCUUCAUUCCCC | GAAUGUUGUCUGGCUCGAGGC | CUCGGACCAGGCUUCAUUCCC |
| 49 | GGGAAUGUUGUCUGGCUCGAGGCACCUGUAACCGGAGAGCAAGAGAUGUUGCUUUCAUCUCUUGUUGUUCUCUUAGGGAUGGAUCUCGGACCAGGCUUCAUUCCCCCCAAUCGUAGCUUCCAAAACCCC | GAAUGUUGUCUGGCUCGAGGC | CUCGGACCAGGCUUCAUUCCC |
| 50 | ACUUCAGGGGGAGAAAGAAGGAGAAAGGAAGCUUUUCCUUUGAGGGGAAUGUUGUCUGGCUCGAGGCAUUUGUAGUAGUUUGAGAUUUAUAGAUCUUACAUAUUCUCUUAUAUUUCGAGAUCUCGGACCAGGCUUCAUUCCCCCCAAUCACAGCUUCCAUGCUUCCAUCUCAGUGAACCCCGACAUC | GAAUGUUGUCUGGCUCGAGGC | CUCGGACCAGGCUUCAUUCCC |
| 51 | AGGGAAUGAAGAUGCUUGCUGAGGGCUUUGCAUGAAGAGGCAGAGAGUGGAAUGCAGCCAAGGAUGACUUGCCGGCAUUGCCUUGUAACAAAAUCUCUUGCGAGUUGCUGGCAAGUUUGUCCUUGGCUACCUUUGGCUCUCUUCUUCUCAUGUGAAGCUCUUGCAUGAUUCAAGCAACGCAUUAGCUAUCACCUU | CAGCCAAGGAUGACUUGCCGG | GGCAAGUUUGUCCUUGGCUACC |
| 52 | AUAUGAGGAUGCUCGUCGAGGGCUUUGCAUGAAGAGGCAGAGAGUGGAAUGCAGCCAAGGAUGACUUGCCGGUGUCUUCUCUUCGAAGGGAGAGUGGUUAGUUUUGGUGUUGCCGGCAAGUUUGUCCUUGGCUACGUUUGGCUCUCUUCUUCUCAUGUGAGGCUUCCGCAUGAUUCC | CAGCCAAGGAUGACUUGCCGG | GGCAAGUUUGUCCUUGGCUACG |
| 53 | GGAACGAAGAUGCUUGUUGAGAGCUUUGCAUGAAGAGGCAGAGAGUGGCAUGCAGCCAAGGAUGACUUGCCGGAAUUGCCUCGAGAUAGCGCCUCGUUUGCAAGUUGCCGGCAAGUUUGUCCUUGGCUACAUUUGGCUCUCUUCUUCUCAUGUGAGGCUCUU | CAGCCAAGGAUGACUUGCCGG | GGCAAGUUUGUCCUUGGCUACA |
| 54 | GGUUAUUUGGCAUUCUGUCCACCUCCAAGUACUGAUCCCUCUCUUAUACAUCCCCAAGGAGAACAACAACUCGAUGGAGGUGGGCAUCCUGCCAACUGACCU | UUGGCAUUCUGUCCACCUCC | AGGUGGGCAUCCUGCCAACU |
| 55 | AUUCCCUUCCACAGCUUUCUUGAACUUCUCCUGUAUAACCCUCCCUCCGACACAGAGGCGGGAGGAGGAAGCGAAGAAGAGGGUCAAGAAAGCUGUGGGAAGGGAUG | UUCCACAGCUUUCUUGAACUU | GGUCAAGAAAGCUGUGGGAAG |
| 56 | GGGUUGCCUCAUAUUUUUCCACAGCUUUCUUGAACUUGAGAUAGGAACAGAAGCUUAAACUAAGCUCAAGAAAGUUGUGGGAAAACAUGGGUAAGCCCA | UUCCACAGCUUUCUUGAACUU | GCUCAAGAAAGUUGUGGGAAA |
| 57 | CGAGCCUCUUCGUUCCUUUCCACAGCUUUCUUGAACUUCCCGUGGGCGCUGGACUCGAGCAAGUUCAGGAAAGCUGUGGGAAUGCAUGGAGAGGUUGGAAUCCCGAGAGG | UUCCACAGCUUUCUUGAACUU | GUUCAGGAAAGCUGUGGGAA |
| 58 | CAUAAAAAGAUGGGUUGCCUCAUAUUUUUCCACAGCUUUCUUGAACUUGAGAUUGAAACGAAAGCUUAAGCUAAGCUCAAGAAAGCUGUGGGAAAACAUGGGUAAACCCA | UUCCACAGCUUUCUUGAACUU | CUCAAGAAAGCUGUGGGAAAA |
| 59 | UGCGAAGUGCCCAAGGGAAUGAGUGAAGCUGCCAGCAUGAUCUAGCUCAGAGACCAAAACCACUCCCCGAGUACCAUUCUGCCAUCUCUAUUGGAGAUGCCAAUGAGUGAAAUUGAAUUGAUGUCAGUCCUGGCUAGCUAGGUCAUGCUCUGGCAGCUUCAUUUGUUCCUUGUUGGACAC | UGAAGCUGCCAGCAUGAUCUA | GGUCAUGCUCUGGCAGCUUCAUU |
| 60 | GGGAAUGAGUGAAGCUGCCAGCAUGAUCUAGCUCUGAAUGACUUGGUGUGUUCAUCAAGGGUUAGUCAUUGCUAGGUCAUGCUGCGACAGCUUCACUUCUUCCCCAUCGAC | UGAAGCUGCCAGCAUGAUCUA | GGUCAUGCUGCGACAGCUUCACU |
| 61 | GAAUAUAUUAAUCCUCAUGCCAACUGCAUGUUUGGAUAUGAACAGAUGAUUCUUUUAGUAGCAGCAGAGCAUUUUGUUAGUCUAUUCAUAUCCAAACAUGGACUUGGCAUGAGGAUUAGCAUAUUCC | CAAACAUGGACUUGGCAUGAG | ACUGCAUGUUUGGAUAUGAAC |
| 62 | GAUCAAAUACAUAGUUCCUAAUUCUAAUUGGCUUACUUCAUCAUGAUGGUAUUGUUUAGGGAGAAUGAUGCUAGUUAGGAUAUGAUGAAGUACCUUAGUGUUUAGAGGCAGUAGAUGUUCA | UGUUUAGAGGCAGUAGAUGUU | AAAUACAUAGUUCCUAAUUCU |
| 63 | GGAGAGGACUUGAAGAAGAAGACAUGGCGUGGUAUUGGAUCGGUUCAUGUCUUAUGAUUGGCUCAACGGGACACGAGCCGAACCAAUAUCACUCAUGUAUUCUUCAUUCACCAUCUCCUCGAAGGUGUCCUCAGUAAUGAUCCA | CGUGGUAUUGGAUCGGUUCAUG | CGAGCCGAACCAAUAUCACUC |
| 64 | CAUGGCGUGGUAUUGGAUCGGUUCAUGUCUUAUGAUUGGCUCAACAGGGUACGAGCCGAACCAAUAUCACUCAUGUAUCCCUCAUUU | CGUGGUAUUGGAUCGGUUCAUG | CGAGCCGAACCAAUAUCACUC |
| 65 | UGCAUCGAUGUCGAUGCUAAGCAACGCCACUUGACAUUUGCAUCAGUAGCCCUCUCGUCGCUCGACAACUACAUCGACGCUGAUGUAAAUGCAUCUGCAUCUACGUCGAUGUAGUUGUCGAGCGAUGAAAGGGUCGAUAUAAAUGUCGAAGGGUGCCGCUCAGCAACUGCGUCGAUGUCG | UCGAUGUAGUUGUCGAGCGAU | UCUCGUCGCUCGACAACUACA |
| 66 | AUGUUCUUCCACGGCUUUCUUGAACUGUGAGCUAUCUUUGCUUCCACUGCUUUCUCGUUACUCCUCAAAAGAGAGAGAGAGAGAUACAUAUCUACAUACACGGAGGAGGUGAAUCAGCGCAAAGUUCAAGAAAUGCGUGGGAAAACAUGGCAGAUCUUUUAG | UUCCACGGCUUUCUUGAACUG | GUUCAAGAAAUGCGUGGGAAA |
| 67 | GUGACAGAAGAGAGUGAGCACCCAUGGUGCUUCUGUGCAUGGUGCAUGGGGGAGUCUAUGCGUGCUCACUGCUCUCUCUGUCAUC | UGACAGAAGAGAGUGAGCACC | UGCUCACUGCUCUCUCUGUCAUC |
| 68 | CGGUGAAGGAGAAUGGGUGUCAUGGAGUUUGGUUGACAGAAGAGAGUGAGCACCCAUGGUGUUUUCCUGCAUGAAGAGGGUUCAUGCUCGAAGCUAUGCGUGCUCACUUCUCUAUCUGUCAACCA | UGACAGAAGAGAGUGAGCACC | UGCUCACUUCUCUAUCUGUCAAC |
| 69 | GCACGCUCAUCUGAGGCCCUGCAUGCGAGGAUGAGGUCACAGUGUGUAGCCAAGGAUGACUUGCCGGCAAGCUUACUCAUGCAUGUUCAUCUUGAAUAGCAGUAGUAGCACGCACCUUUGUUAAAGGCCGGCAAGUCGUUUUUGGCUACAUGUUUGCCUAUUCUUAUCAUGCUAGGCCAAAGA | UAGCCAAGGAUGACUUGCCGG | GGCAAGUCGUUUUUGGCUACA |
| 70 | GAUUUCUUCGUGAUAAGAAGUAUAGAAGAAUCUGUAAAGCUCAGGAGGGAUAGCGCCAUGGAUGAUGUGGAACAUAAAUUCUGAAAGCUUUUGGUGAUUCUAUUUGUCUAAUGCGCUAUCUAUCCUGAGUUUAAUGGCUUCUUCUUACUCAACAAAGACGAAAUCGAUAAAUGAGU | UCAGGAGGGAUAGCGCCAUG | CGCUAUCUAUCCUGAGUUUA |
| 71 | GUAAGGAAGAAUCUGUAAAGCUCAGGAGGGAUAGCGCCAUGGACGAUGAGGAACAUGAAUUCUGAAACCAUUUGGUGGUUCUAUUUGUCUAAUGCGCUAUCUAUCCUGAGUUUAAUGGCUUUUUCUUACU | UCAGGAGGGAUAGCGCCAUG | CGCUAUCUAUCCUGAGUUUA |
| 72 | GAACGUAAGAAUCGGACUGUCGUUGAAAUGGCAAGAAGUUUGCUUAAAGGAAAACAUCUUCCAAAUCAGUUUUGGGCAGAAGCAGUUGCAACAGCAGUUUAUUUUUGAAUAUUUCACCAACAAAGGCUGUUA | AAUCGGACUGUCGUUGAAAUGGCA | UGGGCAGAAGCAGUUGCAACAGCA |
| 73 | UGGAAGGUGGGCUGUGCAGGGUGGAGAAGCAGGGCACGUGCAUUGCUAUCUAACAUCUGAGAGAUAGUUCCUCAUGUGCUCAUCUUCUCCACCAUGCCCAACCC | UGGAGAAGCAGGGCACGUGCAU | UCAUGUGCUCAUCUUCUCCACC |
| 74 | AGGUGGGUUGUGCAGGGUGGAGAAGCAGGGCACGUGCAUGGCUUGCACGUCUUCUUCUUCCACCAACGAAGCCUGUGCCUGUUCUUCAUGUGCCCAUCUUCUCCACCAUGCACAGCCAAUCUUAUUUCACCGCAAGCUC | UGGAGAAGCAGGGCACGUGCAU | UCAUGUGCCCAUCUUCUCCACC |
| 75 | GUGCACCGAGUGGAUGAAGCUGCCAGCAUGAUCUGAUCAUCCCUUGGAUCUGUGUGUCAUGCACCUCCACAGGAGACAAAUCAGGUCAUCUGGCAGUUUCAUCUGCUGAUGGGAGCACC | AGGUCAUCUGGCAGUUUCAUC | AGUGGAUGAAGCUGCCAGCAU |
| 76 | GUGUAGCACCAUCAAGAUUCACAUGUACUGAGUUAUAGCUCUCUGCUGCAAAUGGGAAUCUUGAUGAUGCUGCAUU | GGAAUCUUGAUGAUGCUGCAU | UAGCACCAUCAAGAUUCAC |
| 77 | AAUGUUGUCUGGCUCGAGGUCAUGUGGCGCACAUGCAUGCAUGGUGGGAUGAGCUAUUUGAACUCGGACCAGGCUUCAUUC | AACUCGGACCAGGCUUCAUUC | AAUGUUGUCUGGCUCGAGGUC |
| 78 | CUUCCACAGCUUUCUUGAACUGCUUCCGUUGAAUGGGAGUGAAUACAAUCUCAAUUGCAGUUCAAUAAAGCUGUGGGAGA | UUCAAUAAAGCUGUGGGAGA | UUCCACAGCUUUCUUGAACUGC |
| 79 | UGAUGCUCAAUUCAACUUCUCAUGUCGGCCACCACUGCACGAUCAACAAGAGAUGCUGAUGAUCUCUUGCUAGUCGUGCAGUGGUGGUUGACAUGAGAAGUCGAAUUGGGCGUUAA | UCAAUUCAACUUCUCAUGUCGGCC | UGCAGUGGUGGUUGACAUGAG |
| 80 | GAAGCUUAUCCUUUGAGGGGAAUGUUGUCUGGCUCGAGGUCACUGCUAGUCCUCCACCCUGUGGAAGAGAUCUCGGACCAGGCUUCAUUCCCCCCAGUCAUAGCUUCC | GGAAUGUUGUCUGGCUCGAGGU | CUCGGACCAGGCUUCAUUCCCC |
| 81 | CAUGGUGAGGGGGCUCGAGUAGGAAAGGGCUACCCGUAGACGGAGUCAGAGGACUCGUCACGGCGUGGAGCCACCACUGGGCUACGCCUCACAUGC | AGGAAAGGGCUACCCGUAGACGGA | CGUCACGGCGUGGAGCCACCACU |
| 82 | UGAGUGGGCAUGAUGGAGGUUGACAGAAGAGAGUGAGCACUCAUGGUCUUUUCCCGCAUUCAUCAAGGGUUUUUUAAUGCUCGAAGCUAUGGGUGCUCACUUCUCUCUCUGUCAAC | UGACAGAAGAGAGUGAGCACU | UGCUCACUUCUCUCUCUGUCAAC |
| 83 | CCUGCAGCCAAGGAUGACUUGCCGACAUUGCUCAGUAUCUGCCGUUGUUUCUGCAGCGAAGUAAACUGGAGAAAGAAGAUGUGGGCAAGUUGUCUUUGGCUACAUGU | CAGCCAAGGAUGACUUGCCGA | UGGGCAAGUUGUCUUUGGCUAC |
| 84 | AAGGAGAAUUCCAAAGGGAUCGCAUUGAUCUCUUUAUAUAUCUAUAGUCUCGAGUGAUCAUGCGAUUCCUUUGGAAUUUUCCAUC | UUCCAAAGGGAUCGCAUUGAUC | UCAUGCGAUUCCUUUGGAAUU |
| 85 | AGUUUUGUUGGUGAGGGUGCACCACGAGAAGCUCAAGCUGCCAGCAUGAUCUGAUAACUCCAUCCUCUUUUAUCUUCCGGAUCAUGGAUGAGGACAUGCAGAUCAUGUUGCAGCUCCAUCUACUGGUGGGGGCACC | UCAAGCUGCCAGCAUGAUCUGA | AGAUCAUGUUGCAGCUCCAUC |
| 86 | UGUGAGAGGGCCAUGAGUUUUGAUGGUGAGGGUGGUGCACCACGAGAAGCUCAAGCUGCCAGCAUGAUCUGAUAACUCCAUCCUCUUUUAUCUUCCAGAUCAUGGAUGAGGACAUGCAGAUCAUGUUGCAGCUUCAGCUACUAGUGGGGGCACCAAAACUACGUUUCGAUCCCCAAUUUCUUUCUCCCUCUAAAC | UCAAGCUGCCAGCAUGAUCUGA | AGAUCAUGUUGCAGCUUCAGC |
| 87 | ACUACAGACAUGUAGACUUCUUCUAACCAGAAGAAGAGAUAUAUGGUUAUCACAGCUUAUUUCAUAUGCUCGGCUUAUGACAACAGCUAAUGCUGCCAUCCAUAUGAAAUAAGCAAUGACAACUAUGUUUUCUCUUAUUUUGGUUAGUAGAAGUUCACAUGUCUGUAGUA | UUAGUAGAAGUUCACAUGUCUGUA | AUGUAGACUUCUUCUAACCAGAAG |
| 88 | GUCCAAAGAAGGGACUUGGAUGGGUGGACACAGCUGCUGGUUUAUGGAUUCCACUGCCCUAUUCCAUGUUUUGAGGACAAGGAUGGUGGAUGGUUUGGGAUAGGCUUGUGGUUUGCAUAACUCAGGAGCUGCAUCAGCCCAGCCUGACCCUUAUUUGGAUU | UUUGCAUAACUCAGGAGCUGC | AGGGACUUGGAUGGGUGGACAC |
| 89 | CAUCAAGCUUUCGUAGCUCAGUUGGUUAGAGCACCCGUUUAGUAAGCGGGAGGUCUUGAGUUCGACUCUCAACGAAAGCAUGU | GCUUUCGUAGCUCAGUUGGU | CGACUCUCAACGAAAGCAU |
| 90 | GUCGGUGUUUGUGGGUGGGGCAUCAUCAAGAUUCACAUGGAUUAGACAGCUACGGUGGUUCUACAUGAGAAUCUUGAUGAUGCUGCAUCGGCAAACAAAUGGCUAUCUCAUUCUGAUGUCCACCGCCUUCGACGCUC | GGGGCAUCAUCAAGAUUCACA | CAUGAGAAUCUUGAUGAUGCUGCA |
| 91 | UGAAUGGUAUGUAGGCUGACAGAAGAGAGUGAGCACACAGCGGCAAUUGCACCGAUCCGAUGCCGUUUCUGUUGCGUGCUCACUUCUCUUUCUGUCAGCUCUCUCUAUCUCUCUUCUCCUUCACAAAAGCACAGCACC | CUGACAGAAGAGAGUGAGCAC | GCUCACUUCUCUUUCUGUCAGCU |
| 92 | GUAUGUGGGCUGACAGAAGAGAGUGAGCACACAGCGGCGAUUGCAUCGAUCUGAUGCCGUUCCUGUUGCGUGCUCAUUUCUCUUUCUGUCAGCUCACCCUAUCUCUUUCUCUCC | CUGACAGAAGAGAGUGAGCAC | GCUCAUUUCUCUUUCUGUCAGCU |
| 93 | GUAUGUGGGCUGACAGAAGAGAGUGAGCACACAGCGGCGAUUGCAUCGAUCUGAUGCCCUAUCUGUUGCGUGCUCACUUCUCUCUCUGUC | CUGACAGAAGAGAGUGAGCAC | GCUCACUUCUCUCUCUGUC |
| 94 | GGUUCGCUUGGUGCAGAUCGGGAACGCUUCGCUCCGGCGGCGGAUCCUGCCCUGCGCCAACUGAAUCG | UCGCUUGGUGCAGAUCGGGAA | CCUGCCCUGCGCCAACUGAAU |
| 95 | GGGCGUUGUCAUCAUUACGGCUUUAAGUUUGUCGAAGGCAGUGGAGGCUCUGUCCAACCAUUGGAAGACAUCUUUUUCUAGUAAGGAAGUAAGUGGUGCACUGAUCUUUCCAUAGUUUUUCACGAACUUGCGGUAGUAGCCUGUUAAACCCA | AGUUUGUCGAAGGCAGUGGAGGCU | UAGUAAGGAAGUAAGUGGUGC |
| 96 | AAGUUUGUCGAAGGCAGUGGAGGCUUUGUCCAACCAUUGGAAGACAUCUUUUUUCAGUAAGGAAGUAAGUGGUGUACUGAUCUUUCCAUAGUUUUUCAUGAACUUG | AGUUUGUCGAAGGCAGUGGAGGCU | AGGAAGUAAGUGGUGUACUGAUCU |
| 97 | CUCCGUCUCAUGGAAGCUGUUUACGUAUGAUCCUAUUUGCAGUCUUCCACAGCUUUCUUGAACUGCAUCUGUUCUUCUCACUCUGGGAAAUUGCGGUUCAAUAAAGCUGUGGGAAACUACAGGUGGAGAUCA | UUCAAUAAAGCUGUGGGAAA | UUCCACAGCUUUCUUGAACUGC |
| 98 | GUAGUCUUCCACAGCUUUCUUGAACUGCAGUCAUCAUCCUUUGGAAGUGGGCAUAUACCUCAGUUGCAGUUCAAUAAAGCUGUGGGAAACUACA | UUCAAUAAAGCUGUGGGAAA | UUCCACAGCUUUCUUGAACUGC |
| 99 | CUAUAUGUGUGACUCUCUAGGCCCAAUAUCGAGCUGGUCAUGAGUCAUACAUGUUAGAACUCCUUUUGACUUAGUGAAUUAUUAUCUCCAUAAUAAUUCACUCGACUCAUCGAUUGCAGAUGUACUAGGCCACUACGUCACA | AUCGAUUGCAGAUGUACUAGGCCA | CCCAAUAUCGAGCUGGUCAUG |
| 100 | GAAGAGGGGCUGCGAUGCGCUCGCCACAGGCUGAGGCUGGGUGAGAGUGUGAUGUUGGUACGGCUCAAUCAAAUCAACUGCGAUUUGUGUAGCUAUAUAUAUAGCUUGAUCCGAUUGAGCCGUGCCAAUAUCGCUCUUUGCUUAGUC | CGAUUGAGCCGUGCCAAUAUC | UGUUGGUACGGCUCAAUCAAA |
| 101 | AAUUGUGGUCUUAUGGAGUCCUUGCUGUUCUCUCUCUCUCUCUCUGGAUAAGGCACCAAGAAGGUUUCCUUCGCAGGAGAGAUGAUGCCGCUGUCGUUUAGGGUUCAGAGAACUCGUAGCUUUGGCAGACUGCGGUAUCAUCUACUGCGGAGGAGACAUGG | UUCGCAGGAGAGAUGAUGCCGCU | AUCAUCUACUGCGGAGGAGACAUG |
| 102 | CGUCGUCCGGGUGAUUGGGGUUGACAGAAGAGAGUGAGCACGCAGCGGCGAUCGCAUCGUCGGGGUGCCGUCGCUGUUGCGUGCUCACUGCUCUCUCUGUCAUCCCGAUCUCCCCGGAU | UGACAGAAGAGAGUGAGCACG | UGCUCACUGCUCUCUCUGUCAUC |
| 103 | GUUAAACAUGAUUUUAAGUCUUGUCACAGGAUAUAAAGUGAUUGUAGUCAUUGAUACUACUAUAACUUCUAUUCUGACAUGUACACCUUAGUUAUGUUUAUGUAAAUGAACCCUAAAUUAUAUUUAUAUUAAUAGUAGUCUUUGUGCUACAAUGUUUUAUGCUCUAUGACAGUAGCUAUAUUUAUGCUUUAAUU | AUAUAAAGUGAUUGUAGUCAUUGA | UGUGCUACAAUGUUUUAUGCUC |
| 104 | GAUAAGUGGACACUCUUUCUUUUUCAUCCACAUGUAAGUUGUUGUAAAACAACCUAACAAGUCUUGGAUAGAUGGGUUCACUAAUUU | AACAAGUCUUGGAUAGAUGGGUUC | CACUCUUUCUUUUUCAUCCACAUGUAA |
| 105 | AUCGGGAAGGGAGGAGGGUUGAGAUUUCAUGUGUGGCGAAGGGCGGUUGACAGAAGAGAGUGAGCACGCAUCGGGUUGAAAUGGCACAAGACGGUGCUGUUUCCGCUGCGUGCUUACUUCUCUUCCUGUCAGUCAGCGUUUGUUGUACUUUUCUU | UGACAGAAGAGAGUGAGCACG | UGCUUACUUCUCUUCCUGUCAGU |
| 106 | GGGAGGGCGAGCCGUGCUGGAGAAGCAGGGCACGUGUUCGUGCCAUCGGAACGGUGGCGGCGGCAACGCGUGCCCGACCUCUCCAGCUCGGACUCGCUCCUCCACC | UGGAGAAGCAGGGCACGUGUU | CGCGUGCCCGACCUCUCCAGC |
| 107 | UAAAUCAAACAGGUUUGAUCAGUUAAAGUCGGUUUAGGGUGAUUCCAAACUAACUUGACUAGUUCAAACCUAUUUGA | AGGUUUGAUCAGUUAAAGUCGGUU | CUAACUUGACUAGUUCAAACCUAU |
| 108 | GAUAAGUCGAGGAUGGGCACCGAUGGUUUGUUUGCAAUCUAAACAUGAGGCUCUGUCCGCAUUAAUGGAACCCAACCAUUCUUGUGGGUUCUCAGCUGUGGAAAUGAGGCCUUGUUUGAGA | GUUCUCAGCUGUGGAAAUGAGGCC | UCUGUCCGCAUUAAUGGAACCCAA |
| 109 | UCAAAAGUUAUAUUGGGAUCCCUAUACUUAUGAAAGUGAAACAUUUAACCCCGUUUCUCUUCACGUCGUCGACUUUGCUGACGAAAAUACUGUAAGGUUUACCACUGAGCACUUGUGGUAUUUUCGUCAGCUGAGUCGACGGUGUAAGGAGAAACGGGGUUAAAUGUUUCAUUUUCGUAAAUAGAUAUUCCAAUAUAACUUUUGAG | AGCUGAGUCGACGGUGUAAGGAGA | UUCUCUUCACGUCGUCGACUUUGC |
| 110 | UCCCUUUGUUAUGUCAAAUUUGGUCAUCAACCCAAUGUGCCGAAGUUGAUUCAACCUGCUCCGUUUUUGCAAAAGAAAUCCUAGUCGCUUUUCUGGAUUCGUUAUUCAAGUGUGAUUUCUUUUGCAAAAUUGGAGUAGGUUGGGUCAACUUUGGCAUAUCGGGUUGACAACCAAAUUUGCCGUCAACUUUGGCAC | UUUGCAAAAUUGGAGUAGGUU | CUGCUCCGUUUUUGCAAAA |
| 111 | CUGGGAGGAGCGAGAGGAAGCACAGGGAUUCCUACAAGCUGUGGGGAUGGGGGCAUCGGCAUGCCCAUACCUGUUCUGGCUGAGAGAUGCAGGUUGAUGGUGCUUGCCGAUCCCUCCCAUGCCGAUGGUUUUUAGUAGCUCGUUCCUCUCCCUUGCCCCUC | UUGCCGAUCCCUCCCAUGCCGA | GGGAUGGGGGCAUCGGCAUGC |
| 112 | UGGAGAGAUAGGAGAGGAAUCAACCAUGAAAGGGCUAUUGUGGUGUUGGUCCGGCUCACUCAGAAAGCAUCAGCAAGAAGGCAAAGCAAACGCCUUACAGAUUGCAUUCUGAUUGAGCCGCGCCAAUAUCUCAGCACUCUUUUCAUUCCUCACUCC | UGUUGGUCCGGCUCACUCAGA | GAUUGAGCCGCGCCAAUAUCU |
| 113 | UGUUUGCAGGUGUGGCAUCAUCAAGAUUCACAUGGAUUACACAGUUGUUUUGAUCAGUGAGAAUCUUGAUGAUGCUGCAUCGGCAAAUACGCGAC | UGGCAUCAUCAAGAUUCACAU | AGUGAGAAUCUUGAUGAUGCUGCA |
| 114 | GUUUAAUAUCUGAUACGUGGGUCAUCGACCCACUAUGAUAUUAAAUU | AAUAUCUGAUACGUGGGUCAU | GACCCACUAUGAUAUUAA |
| 115 | GAAGCAAUAGUUGGAUGUCAUCUGGAGUUCCUUCAAACCACUUCAGUGUAGGGUCAUGUUUCGCCUCGGACAUACAUCAGCCCUAACUGAAGUGUUUGGGGGAACUCUGGAUGCCAUUCCA | UGAAGUGUUUGGGGGAACUC | UUCCUUCAAACCACUUCAG |
| 116 | ACUAUGAUCACGUUGGGGGUUCAGAGUUCGACGGAAGUCCGGACGGUCAUCGGAGGUUCUACGGGAACAAAUCCGAGAAGUCCAGGAGCUUGCCAAAGAAGCUCAUCGGAACUCGCCAAGUGGAUCAUCGC | AUCGGAACUCGCCAAGUGGAUCAU | AUCACGUUGGGGGUUCAGAGU |
| 117 | UUGGAUUACCUCUGGAGUCCCUCAAACAAUUCAGUAGGGCUCUUUGAGCUUAAACUUGAGAGCUGGUUCUCACUGAAGUGUUUGGGGGAACUCUGGAUGUCAUCCAACACCUCAAGAACUAUGUGCUAUUGUUUAGAUCAAGCCCU | UGAAGUGUUUGGGGGAACUC | UCCCUCAAACAAUUCAG |
| 118 | CGACCUCCAUUGCACUUGGGAGGGGCGCUGGUCUACCAUCUCCCCAAGUGGGAGAGUGGACGUCGU | CAAGUGGGAGAGUGGACGUC | CUCCAUUGCACUUGG |
| 119 | GAUGGCAAAAGUAUGGAAGCAUCCAUAAAGCUCAGGAGGGAUAGCGCCAUUGAUGACAAGGAAAGACCUGGGAUUGCUCUUUCAUCUGCUGCGCUAUCUAUCCUGAGUUCCAUGGGCUCUUCCUGCUCGC | UCAGGAGGGAUAGCGCCAUU | CGCUAUCUAUCCUGAGUUCCA |
| 120 | CUUGUUGUGACGGAGGUGAUUUUUGGGUAAAAAUCUCCUCUACUUCUCGGAGCAAACCAAUUGUGUUUGUAUAUUCCAAAAGUAAACGUCGCAUAUCAAGU | UGUGACGGAGGUGAUUUUUGGG | AGUAAACGUCGCAUAUCAAG |
| 121 | GUGGUUGGCCAUGGGUUUAACUGUGAUGGGGGUGCACCAUGAGAAGCUGAAGCUGCCAGCAUGAUCUGAGAAGUACUCCAUCCUCCCUGUCUUCUGGAUCUUGGAUGAAGACAUGCAGAUCAUGUUGCAGCUUCAUCUACUGGUGGGAGCACCAAAAGAGCACAGAGAAGACUCAGCCUACUUUUUCUUUCUCCUUUUAACC | UGAAGCUGCCAGCAUGAUCUGAG | CAGAUCAUGUUGCAGCUUCAUC |
| 122 | CAUCAAAAGCAGCUGAAGCUGCCAGCAUGAUCUGAUGGCUGCUCCUUCUUCUCUAUCCUCUCACAACUUCGCAAGGAAUCAAUCAGAUCAUCUGGCAGUUUCAUCUGCU | AGAUCAUCUGGCAGUUUCAUC | AGCUGCCAGCAUGAUCUGAUGG |
| 123 | GUAGCUGAAGCUGCCAGCAUGAUCUGAUCAUAUCCUGGAUCUGUGCGUUGUAUGGAUCUCCACAGGGAGAAAAAAAACUCAGAUCAUCUGGCAGUUUCAUCUGCU | AGAUCAUCUGGCAGUUUCAUC | GAAGCUGCCAGCAUGAUCUG |
| 124 | GUUGGCCAUGGGUUUAACUGUGAUGGGGGUGCACCAUGAGAAGCUGAAGCUGCCAGCAUGAUCUGAGAAUUCCUCCAUCCUCUCUAUCUUCUGGAUCUUGGAUGAAGUCAUGCAGAUCAUGUUGCAGCUUCAUCUACUGGUGGGAGCACCAAAAGAGCACAGAGAAAACUCAGCCUACU | UGAAGCUGCCAGCAUGAUCUGAG | CAGAUCAUGUUGCAGCUUCAUC |
| 125 | AGGACGAUGGUCGCUCUAUAUAGUUCUGCAACUACAUUAGCAUUGCUCUGCAUUUGCAUCAGUGGGAGGUGAGGCAAAGGCCUUAUAUCCCGUCGACACAGAUACAAUGUGACGUGAUGUCAAUGCAUAUACCUCACCUCGCGUCGAUGCAAAUGUAGAACAGUACUGAAGAAGGAAGAGGAGGGAGAGCAAUAAGAGGAGAGUGUCCC | AUGUAGAACAGUACUGAAGAAGGA | AGGCCUUAUAUCCCGUCGACACAG |
| 126 | GUGACCACAACAUGAGAAACGGCGCUGAGCGAUUUCGUUGGAAACGCCCAAAGCCGUUUCUCAUGGUGUGGUCACC | UGACCACAACAUGAGAAACGG | GUUUCUCAUGGUGUGGUCACC |
| 127 | GAACAGCUACGAGGGUCGGAUGAAGUCGGAGACGAAGGAGGUCGUGAAGAAGAGGUGAAAUGGACUGUGCCUGGCUCCCUGCAUGCCACUCGUAUCGACGCCAAUCAUACCCAUGGAUUGGCAACUGCGGCUGGCGUGUGAGGAGCCAAGCAUACCUCUCACUCUCUCUCUCUCUCUCUCUCUCACUGAGACCUUUGUUCUCUAUUCU | UGCCUGGCUCCCUGCAUGCCA | GCGUGUGAGGAGCCAAGCAUA |
| 128 | CGACGAUAUGUCUAAUGCCAAUCGCGUAGCUGGGCAGACUUAACUUCAUGGACUAGGCUAAGCCUCACUUAUAAGUUGGGCUGAGCCUUACCAUUGAACUAGGUCAUGCUUGGCUCGCGA | ACUUAACUUCAUGGACUAGGCUAA | AGCCUUACCAUUGAACUAGGUCA |
| 129 | GUUGGGUAGACUUAACUUCAUGGACUAGGCUAAGCCUCACUUAUAAGUUGGGUUGAGCCUUACCACUGAACUAAGCCAUGCUUGGCU | ACUUAACUUCAUGGACUAGGCUAA | AGCCUUACCACUGAACUAAGCCA |
| 130 | GUACAUCGUAUCGUUAAGUUCUUUAUGUUGUUUCUCGCGUAUGCUAUCGUUACCGUGAAGCGUACGAUAUAAAGAUCGUAACGGCACCUUGUACG | UAUAAAGAUCGUAACGGCACC | AUCGUUAAGUUCUUUAUGU |
| 131 | CCGUGUCGUUACGUUCUUUAUAUUGUCGCUCUAGUGUACUAUCGAAGCCUACAAUAUAAAGAUCGUAACGGCACCGU | UAUAAAGAUCGUAACGGCACC | GUCGUUACGUUCUUUAUAU |
| 132 | CCGUCAUGGCACAAUCUCCGAGACUAUGUUAGGUGGUGGUACCGUCCAGUGGUAGGGUGUCAGGCAAUGGUAACCACCACCCAAGCAUAAUCUUCGAGAUUGUGUCAAGCGGU | AUCUUCGAGAUUGUGUCAAGCGGU | GUCAUGGCACAAUCUCCGAGACU |
| 133 | AGUCACCGAUGAGUCGUCAUUAGCGCUCGAUGAACUUGAGCCCAUCGAACCUCGGCGCGAACUUGUCUUCCAUACUCUUUCUCGAUCCUGAUCUGGCACCGCCCUCCCUUGACAAUCUUGAUCCCGACGUGUUCAAGUUCAUCGAGACACUAGUGACGGCGCAUUGGUGACUU | UCGAGACACUAGUGACGGCGCA | GUCGUCAUUAGCGCUCGAU |
| 134 | AUAAAUCACCUUGAAAUAGUUACUGUGAUGUUGGCUCGGUUCACUCGGAGCUCGGUUACUGCAAGGAAUGUCUUCAGACCUUUUUUCUGAUUGAGCCGCGCCAAUAUCUUAGUACU | UGUUGGCUCGGUUCACUCGGA | GAUUGAGCCGCGCCAAUAUCU |
| 135 | GGAGAAGAGAGAGAGUACAGCCUCGGCAUGCCGCCAGUGAGAGUCCCAGGUGAAGGCUGUACCCUCUCUCUUCUUCU | AGAAGAGAGAGAGUACAGCCU | GCUGUACCCUCUCUCUUCUU |
| 136 | AUGAGAGUUGUGGGUUCCGGCGUUCUUGCGGGGGCGUCGUUGGUUGACAACGAGAGAGAGCACGCCGGUUCGCAAGCUAUGGCGGCGGCCAUGGUUGUGCACUAGCGUGCUCCUUCUCGUUGUCACACGCGGUCGCCUCACAAGGCAGCUUCUCCUGCUCGCCCUCUUUUUCUUCCUUGG | GUGCUCCUUCUCGUUGUCACA | UGACAACGAGAGAGAGCACGCC |
| 137 | ACCGGGUGGUGGAUUACUGGGUGAACUCUCCUUUGGCAGAUGGAAGAAGAUGCAACCCGAAUUCUUGCUGCCAAAGGAGAAUUGCCCAGCAAUCCUAAUCCAGUU | UGCCAAAGGAGAAUUGCCCAG | GGGUGAACUCUCCUUUGGCAGA |
| 138 | CAAGUGAAAGACCCAAACAGUAAACCAAUAGGUGGAACUAUCGUGGUACAAUCUCCGAGACUGUGUUAGGUGGUGAUACCAUCGAUCUGGGUGGUGGUACCACCCAGACUCAAUCUCUGAGAUAGUCUGGCAGUGGUACCGCCCAAUGUCAGUGCUG | AUCUCCGAGACUGUGUUAGGUGGU | CACCCAGACUCAAUCUCUGAGAUAG |
| 139 | UGGGUGGGUGGGGAGGUCUGUGUAUGUGAAGUCUGUAAAUGAUCCUCUUUGUAGUUUUCCACAGCUUUCUUGAACUGCAUCGGUUGCUCUAUGACGGAGGCUUAAGCUGCGGUUCAAUAAAGCUGUGGGAAGUCACAAAGAGAGACCAAAUUGAGAAGCGUAUGCAUGGAGAUCUUUCUGCUGCUUUC | UUCAAUAAAGCUGUGGGAAG | UUCCACAGCUUUCUUGAACUGC |
| 140 | CAUCAGACGUGACGGUUGGACGGUUCCUCCAUCCGCUAUUGGGAGGAACGUGUCCCCACUUUGGCGGGUGAGGGACACCACUCCAUCCGCUGUUGG | AGACGUGACGGUUGGACGGUUCCU | GGACACCACUCCAUCCGCUGU |
| 141 | AGGCUGGGCGGUGGUACCGCCCCUGUCAGGCGGUGGCACCGCCUGGGCUCAGUCUCCGAGUGAGACUGGGCGGUAGCACCGCCCCUGUCAGGCGGUGGUACCGCCCGAGCUCGGUCUC | AGUGAGACUGGGCGGUAGCACCGC | GUGGCACCGCCUGGGCUCAGUC |
| 142 | GUGGCAUCAUCAAGAUUCACACCGACCGGAUGCCCCGCCGUCCUCGUCGUCGAUGAGAAUCUUGAUGAUGCUGCA | UCGAUGAGAAUCUUGAUGAUG | UCAUCAAGAUUCACACCGACC |
| 143 | GUUGCACCGCCUGGCAGAGCUCGAAGACUGAGCCUCUAGGCGGUGCCACCUCUUGUCAGGGGCGGUUGCACCUCUUGCCAGAGCUCGAAGACCGAGCUCAGGCGGUGCCACCUCCUGACUGGGGCGGUUGCACCACCUGGCAGAGCUCGAAGACUGAGCUCCAGGCGGUGCCACC | UCGAAGACUGAGCCUCUAGGCGGU | ACCUGGCAGAGCUCGAAGACUGAG |
| 144 | GGUGUGAGGAGUUGACGGUUGUUGUCCUGUAAUGAUCUCGAAGGGGCUCUUGUUGGAUGCAGAGCUCCGCUGCAAGUUGUAGGAGAAUUGGGCAAUGUCCAACAGCUUCACCCA | AGGAGUUGACGGUUGUUGUCCUGU | UGGGCAAUGUCCAACAGCUUCA |
| 145 | CGAUGAAAGAGUUAUUCACUAUGUUAGCAUCAAUGUAGUUGUCGAGCGACAAAGGGCCACUCGACGUUGACAUUGAUGCAGAGUGAUGUCGCUUUGCAUAUGUGUCGACAUCGAGUGGCAUUUUUGUCGCUCGACAAUUAUGUCGAUGCUGAUGUAGUGAGUGGCCCUUUCGUCGC | AGCAUCAAUGUAGUUGUCGAGCGA | UGUGUCGACAUCGAGUGGCAU |
| 146 | GGCGGUAGCACCGCCCCUGUCAGGCGGUGGCACCGCCUGGGCUCAGUCUCCGAGUGAGACUGGGCGGUAGCACCGCCCCUGUCAGGCGGUGGUACCGCCC | AGUGAGACUGGGCGGUAGCACCGC | GUGGCACCGCCUGGGCUCAGUC |
| 147 | CCUCCCAUUGGCAAGGCCUCUUGCUCAAGUGAGUAUUCCAUUCGCCGCCUCCCCCGGGCCUCGCCAAUGAAAUACUCACCUGGGCAAGAUGCAUUCCGUGGAAGGA | UCUUGCUCAAGUGAGUAUUCCA | AAAUACUCACCUGGGCAAGAUG |
| 148 | UUAAAAUUAAAUAGUUAUUUUUGUCAUUUUAAAAUUAAAUAAUUAAUUCAGUAUAAUACUGUAGCACUGUAACGGUACCGGGCGGUCCGUGUACCGGUAAUCUGUCGGACCGGUACGUACCGCCCGUACCGAUCGGUACGCUUCGGUAUGACAGACCUUGUUUAGUUGUUUAUGACAUCUUUUAUAAAAAUCAUAAUAUCUAGAACUUUAAA | AAUACUGUAGCACUGUAACGGUAC | ACCGAUCGGUACGCUUCGGUAUGAC |
| 149 | CUUUGAAGUAGUGGAAUGUGUCUAUGCUACACUAUACUAUCUUUUACUUUAUUUUAUUUUUUUUUUGCAAAAAGACAAUAAUAAUAUUAUGAUAAAGUAAAAUAUAGUUUAUUGUAGCACGAACACGCUCUACUAUUUCAAAGC | UUUGAAGUAGUGGAAUGUGUCUAU | GAACACGCUCUACUAUUUCAAAGC |
| 150 | GAAGUGGGUGUUUGGAAUGAAGUUUGAUCCAAGAUCCUUCCACAAGCUUUCCCUCUUUAUAUGCUAUGGGGUUUCUUGUUUGGUUUGGAUGUGAUCUCGGACCAGGCUUCAUUCCACACACCUUAAUUUCC | UCGGACCAGGCUUCAUUCCAC | GAAUGAAGUUUGAUCCAAG |
| 151 | GGCAUAGUCUCCGAGAUUGUGUUAAGCGGUGAGACAACCGGUCUGGUAGUGGUAUCACCCAAACAUAAUCUCCGAGAGACUGUCA | UCCGAGAUUGUGUUAAGCGGU | CCAAACAUAAUCUCCGAGAGA |
| 152 | GAUUUCAUCAAUUCGGAGUGAUCACGGUGGUGAAUUUCAAAACCGUGAUUUCCAAAAUUUUUGUGAAGUUA | UCAAUUCGGAGUGAUCACGGUGGU | AACCGUGAUUUCCAAAAUUUUUGUG |
| 153 | CUAAAUUGAACCUAAUCUAGUCCAAAUUAUACUAGUCUAGGGUGGUUCCAGACCAGUUAAAUGAGGAUUAGAGAUCGGUUCAGUUAGG | UUAAAUGAGGAUUAGAGAUCGGUU | CUAAUCUAGUCCAAAUUAUAC |
| 154 | GGGGUGUGAGAAUGACAGUGCGGUUCUUCUUUGGCAAGGAGUCGAAGCUGGUCCAAAUGCAUGCUUAAUUCCCCUCCUUGCCAAAGGAGAAUUGCCCUGCCAUUCGCUGCCCCCGCAGUCGAUCGCUC | GUGCGGUUCUUCUUUGGCAA | UGCCAAAGGAGAAUUGCCCUG |
| 155 | UCCUGUAUGAGUGCUCCACUCUUUGAUACCGGACUUAUAGGUUUGGAUGUUCCAGAUCUAGCAUAGUCGGUCAUCGGGAGUGGUAGCCAACUUUACGAGGACUAUUGAAUGUCGAUAGAGGAUUAUCUACUCUC | AGAUCUAGCAUAGUCGGUCAUCGG | AUACCGGACUUAUAGGUUUG |
| 156 | UCCUGUAUGAGUACUCCACUCUUUGAUACCAGACUUAUAGGUUUGGAAGUUCCAAAUCUAGUAUAGUCGGUCAUUGGGAGUGGCAG | AAAUCUAGUAUAGUCGGUCAUUGG | AUACCAGACUUAUAGGUUUG |
| 157 | UUGGGAUCGGCCGGGGGUAUUUAUACCUGCGUGCGGGAGUCGGUUGCGCCCCGGUUCGGCAUGGCCGUUGACUCUCGCGGGGGGGGAUAACCCC | AUACCUGCGUGCGGGAGUCGGUUG | GCCGUUGACUCUCGCGGGGGGGGAUAA |
| 158 | GUGGUGGUACCACCUAGUGGCAGUGUGUUAGGCGGUGGUACUGCCCAAUGUCAGUGCUGUAGGUGGUGGUACCGCUAGUACCCCAAAAACUAGGG | AAUGUCAGUGCUGUAGGUGGUGGU | ACCACCUAGUGGCAGUGUGUUA |
| 159 | GUGGCCGGGGUCAUUUGGGUUGACAGAAGAGAGUGAGCACACAGCGGACGGUGGCAUCGAUCCGCGGGAUGCCGUCGCUGUUGCGUGCUCACUCCUCUCUCUGUCAUCCCUGUCUCCCUGGCGUUGCCUGCAGAGAGAGAUGCGC | UUGACAGAAGAGAGUGAGCACA | UGCUCACUCCUCUCUCUGUCAUCC |
| 160 | GUACCGCCCAACAUAGUCUUCUAGGUCAUGGUACCACCAAACUAGGUGGUGGUAUCACCCAGUGUCAGACUGUAGGUGGUGGUACU | AGUGUCAGACUGUAGGUGGUGGUA | CGCCCAACAUAGUCUUCUA |
| 161 | CCGGCGUCGUCCCGGGUGAUCUACUUGACAGAAGAGAGUGAGCACACAGCGGCGUGGGUAUCGCCGGGAUGCCGUCGCUGUUGCGUGCUCACUUCUCUCUCUGUCAUCCCCGUCUCCCUGGCGGUGCCGGA | UUGACAGAAGAGAGUGAGCACA | UGCUCACUUCUCUCUCUGUCAUCC |
| 162 | GCGGUACCACCGCCUGUUCUGGGUGGUACCACCGCCUACAGUCUGACACUGGAUGGUACCACCACCCAGUCUAGUGGUACCACCGCCUAGAAGACUGUGCUAGGUGGUACCAUUUCCCAGACUGGUGGUACCACC | AGAAGACUGUGCUAGGUGGUACCA | UGACACUGGAUGGUACCACCA |
| 163 | UUGAUGUCGGUUCUUGAUGCGUUACCUGUCCCCUUGGGUGGCUUGAUGCGGGUCCUUCAUUAAACCAAAAGAAAUUAUACCAAACGAUCAUCCCAAGAGGGCAGGUAUUGUAUCGGUUCUUGACAUCAAGAUACCCAAGAUGACAGUCAUCGCAUCAAGAAACGAUAACAAGACACCCAAGAGGACAGGCAUCGCAUGAGUAAUCUCCAUCAGA | UUGAUGUCGGUUCUUGAUGCG | UGACAGUCAUCGCAUCAAGAA |
| 164 | UGCGAAGGGUGCAUCACUGGGCACUCUCUUCUUUGGCAGUGGCAACAACGCCGGCUUCCAUGCGACGUACCGGUGCAGCUGAGCCCACUGCCAAAGGAGAUUUGCCCAGUCAUUCAUUCACCUUCGCUACCUACUUCUUUCAACAC | UGGGCACUCUCUUCUUUGGCAG | ACUGCCAAAGGAGAUUUGCCCAG |
| 165 | AGGUGAGGGCCGCUCUGCAUCUGUGUUGGCACCAACGUUGAUGCAAAGCAACGACCGAGCGGCCCUUUUGUCUCUCGACAAUUGCGUUGGCGUCGACGCAGAUAUAGAGCAGCCCUCACCUC | CGACGCAGAUAUAGAGCAGCCCUC | UGAGGGCCGCUCUGCAUCUGU |
| 166 | GAAGUUUUUCUUUGAGGGGAAUGUUGUCUGGCUCGAGGACACUUUCGACUCACCUCUCUGUGGAAGAGACCUCGGACCAGGCUUCAUUCCCCCCAAUCACAGCUUCCAUCUC | GAAUGUUGUCUGGCUCGAGGA | CCUCGGACCAGGCUUCAUUCC |
| 167 | GCGGGUGUAGCAUCAUCAAGAUUCACAUGAGUCGAGCGGCCGCGUUACGAUUUCAAGUGAGAAUCUUGAUGAUGCUGCAUCGGCAAUAAGCGACUAA | GUAGCAUCAUCAAGAUUCACA | AGUGAGAAUCUUGAUGAUGCUGCA |
| 168 | CGCUUAAUCUGACGGUACUAUCGCCUGACACAGUCUAGGAGACUAUGUUUGGGUGGUACCAUUGCCUAGUCUAGCGGUACCACUGCUUAACAUAGUCUUAAAGACUGUGUCUAGGCGGUACCAUCGCUAGACCGG | AAAGACUGUGUCUAGGCGGUACCA | AGGAGACUAUGUUUGGGUGGUACC |
| 169 | GGAGAAACUAAAUGAAAACUCAAGAGCUGCAGCAUAAGUUUGAGAAGAGAAGGAAGAGGAAUUGAAGUAGAGGAAGAAAAAGCUUUGCUUUGGCUUGAGAUUUUGCAUCAAUUCCACACCUUUGGGACUCAAAUUUGU | AGAGGAAUUGAAGUAGAGGAAGAA | UUGCAUCAAUUCCACACCUUUGG |
| 170 | CUGGGCGGUGCCACCGCCUAGCUCUCCGGUGCUGGGCGGUGCCACCGCCUGGCUCUCGGGUGCUGGGCGGUGCCACCGCCCAGU | UGGCUCUCGGGUGCUGGGCGG | CCUAGCUCUCCGGUGCUGG |
| 171 | AGGACCAUUCCUAGACCUAUGAGUUUCUUUAUCCACUCAAUUAUCUUUAGGACAUGGUUCUG | ACUCAAUUAUCUUUAGGACAUGGU | AUUCCUAGACCUAUGAGUU |
| 172 | GCCUCUCUUAUGGGCUGAAGGGGGAGGUUGUUGGGCAGACAUCUCAUUACUUCAGCCCAUAAUGGGCU | UCUUAUGGGCUGAAGGGGGAGGUU | UCUCAUUACUUCAGCCCAUAAUGGG |
| 173 | AGGAAGGAAGACGCCGUGAAGAGGGUGGGUGUGAGAAUGACAGUGCAGUUCUCCUCUUGGCAAGGAGUCCAAGCAGGUGCAGAUGCAUGUGCCUCCUCCCUCUCUUGCCAAAGGAGAAUUGCCCUGCCAUUUUCCCCCACUGUC | UGCCAAAGGAGAAUUGCCCUG | UGCAGUUCUCCUCUUGGCAA |
| 174 | UUGACAUGCGAGAGAUCUAAGCAUGUUAGUUGAACCACUAACGUAUAAGAUUUUAGAGCAUGUCGAC | UAUAAGAUUUUAGAGCAUGUCGAC | GACAUGCGAGAGAUCUAAGCA |
| 175 | GAGGUUGCACCGCCCAGUCUCGCUCGGAGACUGAGCCCUGGGCGGUUGCACCUCUUGGC | ACUGAGCCCUGGGCGGUUGCACCU | UUGCACCGCCCAGUCUCGCUCGGA |
| 176 | CCUCAAUCUGAAGACAUGGCUGGAUAUGCUCAUGCAGCCAUGUAGUUGGAUUGAGCCGC | CCUCAAUCUGAAGACAUGGCUGGA | GCCAUGUAGUUGGAUUGAGCC |
| 177 | AUUCCGAGGAUUGGCCGAGCGAAUAAGUAAACUUCAAAGUGGAUUUGUUGUGCGAAGAGUUCGUUAUGAACCGUGGCCGCUUCCCUGGGGUGGGGGCGGCUCCUCCGUU | GAGGAUUGGCCGAGCGAAUAAGU | AAAGUGGAUUUGUUGUGCGA |
| 178 | GACUAUGUCAAGCGGUGGUACUACCUAGUGUCAGUGUCAGACUAGGUGGUGAUACCGCUUGGUGUUAGACUGACAAGAGGUGGUACCGCCAGUUGUUAGUCU | UUAGACUGACAAGAGGUGGUA | UCAGUGUCAGACUAGGUGGUGA |
| 179 | AGACUGUGUCAAGCGAUGGUACCACCAAUCUGGGUGGUGGUACCACCCAGACUCAGUCUC | AGACUGUGUCAAGCGAUGGUACCA | GUACCACCCAGACUCAGUCUC |
| 180 | CGACAUUGUUCUGAGCUUUCCGAGGCUAUUGUGUGAUGCGUGACAUCAUCCUAUAUAGCCUCGGAUGGCAUGGAAGCAGGUGGUCGC | AGCCUCGGAUGGCAUGGAAGCAGG | GUUCUGAGCUUUCCGAGGCUA |
